# Synthetic communities of maize root bacteria interact and redirect benzoxazinoid metabolization

**DOI:** 10.1128/msphere.00159-25

**Published:** 2025-08-25

**Authors:** Lisa Thoenen, Christine Pestalozzi, Tobias Zuest, Marco Kreuzer, Pierre Mateo, Mikiko Karasawa, Gabriel Deslandes, Christelle A.M. Robert, Rémy Bruggmann, Matthias Erb, Klaus Schlaeppi

**Affiliations:** 1 Institute of Plant Sciences, University of Bern 27210, Bern, Switzerland; 2 Department of Environmental Sciences, University of Basel 27209, Basel, Switzerland; 3 Department of Systematic and Evolutionary Botany, University of Zurich 27217, Zurich, Switzerland; 4 Interfaculty Bioinformatics Unit, University of Bern 27210, Bern, Switzerland; University of California Davis, Davis, California, USA

**Keywords:** plant root bacteria, root exudates, benzoxazinoids, synthetic community, metabolization

## Abstract

Plant roots are colonized by diverse microbial communities. These communities are shaped by root exudates, including plant-specialized metabolites. Benzoxazinoids are such secreted compounds of maize. Individual microbes differ in their ability to tolerate and metabolize antimicrobial benzoxazinoids. To investigate how these traits combine in a community, we designed two synthetic communities of maize root bacteria that share six common strains and differ in their ability to metabolize benzoxazinoids based on the seventh strain. We exposed both communities to the benzoxazinoid MBOA (6-methoxybenzoxazolin-2(3H)-one) *in vitro* and found that the metabolizing community did not degrade MBOA to its aminophenoxazinone, as observed for individual strains, but, as a community, they formed the corresponding acetamide. MBOA shaped the differential compositions of both communities and increased the fraction of MBOA-tolerant strains. The benzoxazinoid-metabolizing community showed a higher tolerance to MBOA and was able to utilize MBOA as their sole carbon source for growth. Hence, bacterial interaction results in alternative benzoxazinoid metabolization and increases community performance in the presence of these antimicrobial compounds. Future work is needed to uncover the genetics of this metabolic interaction and ecological consequences for the bacterial community and the host plant.

**IMPORTANCE:**

We investigated how maize root bacteria—alone or in community—tolerate and metabolize antimicrobial compounds of their host plant. We found that the capacity to metabolize such a compound impacts bacterial community size and structure and, most importantly, benefits community fitness. We also found that interacting bacteria redirected the metabolization of the antimicrobial compound to an alternative degradation pathway. Our work highlights the need to study the teamwork of microbes to uncover their community traits to ultimately understand the ecological consequences for the bacterial community and eventually the host plant.

## INTRODUCTION

Most multicellular organisms are closely associated with microbial communities, which provide important functions to the host. Plant microbiomes function in promoting growth, providing nutrients, and protecting from disease, but some microbiome members can also act as pathogens (1–3). The members of a microbiome interact with each other, for instance, when colonizing the roots of a common host plant (4). Root exudates, which can account for up to one-fifth of the plant’s assimilated carbon (5), contain primary metabolites, including sugars, amino acids, organic acids, and fatty acids, as well as secondary metabolites. The latter, also called specialized metabolites, act in diverse ways to govern the interactions with the plant’s environment. Often, these compounds have antimicrobial function, selecting adapted microbes and thereby shaping the species-specific root microbiome (5–9). Examples include glucosinolates, camalexins, triterpenes, and coumarins in *Arabidopsis thaliana* (6), the saponin tomatine from tomato (10), and benzoxazinoids (11–15), diterpenoids (16), zealexins (17), and flavonoids (18) in maize. While there is increasing knowledge of how individual microbes react to these compounds, it is largely unknown how they cope as communities with antimicrobial root exudates.

For interacting with each other, microbial community members have, on one hand, developed mechanisms to optimize their fitness through competitive mechanisms, such as the production of antimicrobial compounds (19). On the other hand, some also adapted to profit from each other to efficiently use resources and accomplish tasks that are not feasible as individuals (20, 21). For example, community members might exchange metabolites to complement each other’s biosynthetic pathways (22). Such interactions not only improve community fitness but also alter final product distribution and give rise to new phenotypes without genetic modification (23). Microbes often cooperate to degrade antimicrobials or pollutants (21, 24) or metabolize complex substrates (25). While there are examples of how microbial communities cooperate to degrade antimicrobial compounds and pollutants, little is known about how root microbiome members interact to cope with antimicrobial plant metabolites.

Crops belonging to the family of sweet grasses (*Poaceae*) include maize, wheat, and rye, all of which produce benzoxazinoids as specialized metabolites (26, 27). These indole-derived alkaloids mainly function in protecting the plant from insect pests and pathogens (26, 28, 29), providing support in iron uptake (30) and structuring the root microbiomes by acting as selective antimicrobials (8, 11–15). Fig. S1 contains the full names and structures of all abbreviated compounds mentioned in this study. DIMBOA-Glc is the main root-exuded benzoxazinoid of maize (13), and its chemical fate in soil is well understood: upon exudation, DIMBOA-Glc is deglycosylated by microbe- and/or plant-derived enzymes (29) to form DIMBOA, which spontaneously converts to more stable MBOA (31). In soil, MBOA has a half-life of a few days, and when further metabolized by microbes to reactive aminophenols (29), MBOA can take three different routes (32, 33): route (I) is favored under aerobic conditions forming aminophenoxazinones, such as AMPO, through oxidation; route (II) results in acetamides, such as HMPAA, through acetylation; or alternatively, route (III) yields malonamic acids, such as HMPMA, through acylation. Route I is probably the most relevant for the rhizosphere, and the resulting AMPO remains detectable in the soil of maize fields for up to a few months (31). Several bacterial isolates were shown to metabolize MBOA to AMPO, which is mediated by the lactonase BxdA (34). Route II was demonstrated for endophytic fungi (*Plectosporium tabacinum*, *Gliocladium cibotii*, and *Chaetosphaeria* sp.) that were isolated from *Aphelandra tetragona*, which metabolized HBOA to various acetamides (32). Following Route III, the maize seed endophytic fungus *Fusarium verticillioides* converts BOA to the malonamic acid HPMA catalyzed by a metallo-β-lactamase (35). There is first evidence that benzoxazinoid metabolization routes vary when different microbes interact. The fungus *Fusarium verticillioides* was co-cultured with the bacterium *Bacillus mojavensis* in the presence of BOA, and metabolization was redirected from HPMA to the aminophenoxazinone APO (36). Hence, benzoxazinoid metabolization in complex microbial communities warrants further research, for instance, to test if microbes metabolically redirect benzoxazinoid degradation.

To study the mechanisms governing composition and functions of microbial communities and their interactions with the host or the environment, synthetic communities (SynComs) are a useful tool (37). A SynCom is a microbial community created by rationally mixing strains from a collection from a specific environment. Strains can be added, eliminated, substituted, or individual strain functions manipulated, thereby allowing the detailed study of SynCom members, their functions, and the resulting community performance (38). Although these reduced systems do not accurately represent nature, they allow the replication of phenotypes mediated by the microbiome.

Here, we aimed to test if maize root bacteria interact to tolerate and metabolize the benzoxazinoid MBOA in small SynComs. We investigated (i) the type of degradation metabolites produced by SynComs and which of its members are responsible for metabolization, (ii) how the ability to metabolize benzoxazinoids affects community growth and tolerance, and (iii) if MBOA shapes the composition of the two SynComs differently depending on their ability to metabolize MBOA. To address these questions, we designed two SynComs consisting of six common core strains and one variable *Microbacterium* strain that differed in the ability to metabolize MBOA. We exposed the SynComs to MBOA and measured community growth, community tolerance, community composition, and benzoxazinoid metabolite profiles. We found the SynCom containing the benzoxazinoid metabolizing *Microbacterium* to convert MBOA to HMPAA, to grow on MBOA as a sole carbon source, and to be more tolerant to MBOA. Interestingly, the benzoxazinoid metabolite HMPAA was not formed by single strains but only in the SynCom in combination of *Microbacterium* LMB2 with other strains. Our results revealed that maize root bacteria interact to metabolize and tolerate benzoxazinoids, which is beneficial for microbial growth.

## RESULTS

### In community, the bacteria metabolize MBOA to HMPAA instead of AMPO

**Fig 1:**
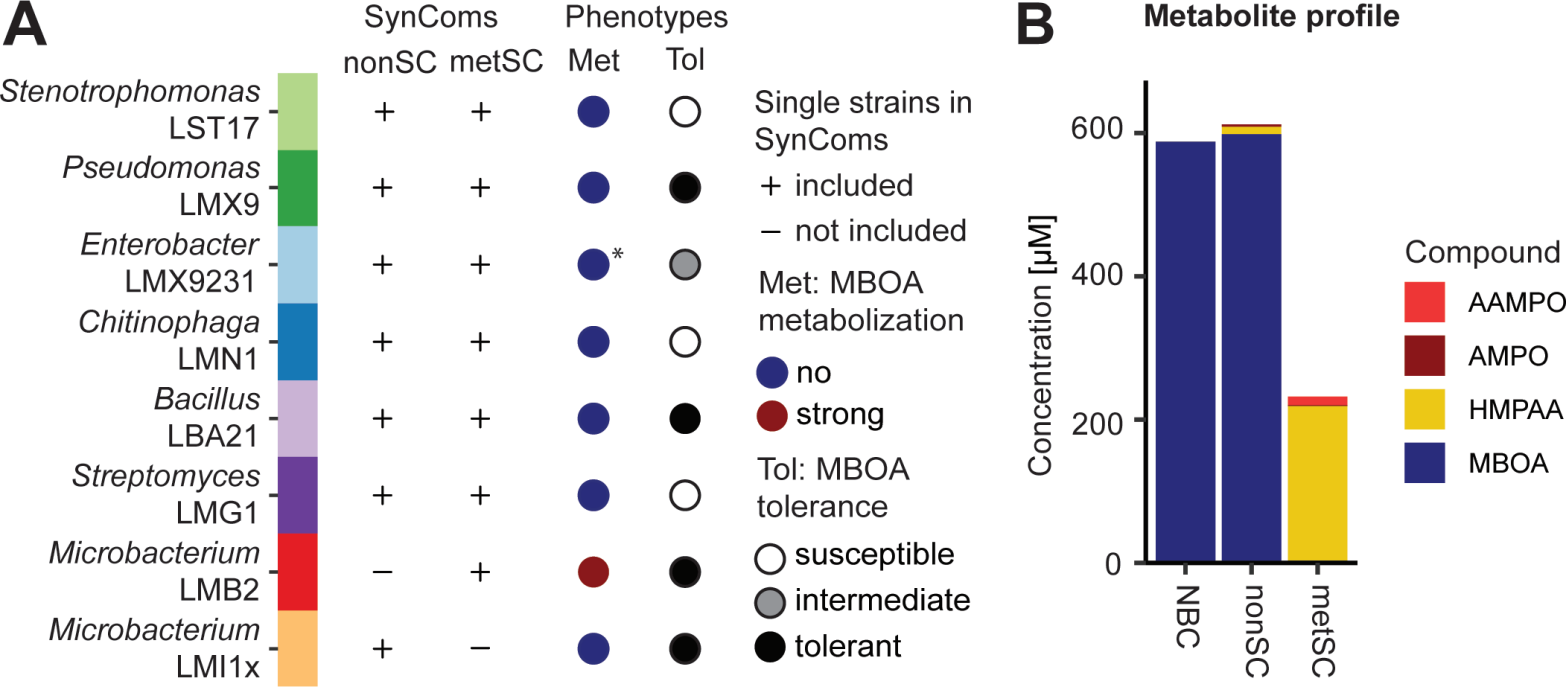
SynCom design and metabolization of MBOA. (**A**) List of bacterial strains selected for the non-metabolizing SynCom (nonSC) and the metabolizing SynCom (metSC) indicated with a “+” if included and a “−“ if not. The phenotypes of each strain in MBOA metabolization (Met, data from our previous publication 34) and MBOA tolerance (Tol based on 2,500 µM data from Fig. S2) are listed in columns three and four. (*) LMX9231 does not efficiently metabolize MBOA to AMPO in liquid culture; see notes in Supplementary results. (**B**) The nonSC and metSC SynComs were grown in 96-well plates containing 50% TSB supplemented with 500 µM MBOA for 68 h. Afterwards, the metabolization of MBOA to AMPO, to AAMPO and/or HMPAA was determined. SynComs and their no bacteria controls (NBC, media with MBOA but without bacteria) were grown in triplicates and pooled before measurements (*n* = 1).

In previous work, we characterized individual maize root bacteria for their abilities to tolerate (8) and metabolize benzoxazinoids (34). In this study, we investigated how these two traits function in community context. To this end, we examined growth and composition of synthetic communities (SynComs) of maize root bacteria and the metabolites they produce after exposure to MBOA. We constructed two seven-member SynComs consisting of taxonomically distinct bacteria that differed in their tolerance to MBOA (Fig. 1A; Fig. S2; see note on SynCom assembly in Supplementary Methods). Both SynComs shared a common core of six bacteria that do not degrade MBOA in liquid culture (see note on LMX9231 in Supplementary Results, Fig. S3). To complete the communities, *Microbacterium* strains, differing in their ability to metabolize MBOA, were added to each SynCom. This resulted in a *non*-metabolizing SynCom (nonSC) containing *Microbacterium* LMI1x, which is unable to metabolize MBOA, and a MBOA-*metabolizing* SynCom (metSC) containing the MBOA degrader *Microbacterium* LMB2 (Fig. 1A). Importantly, monocultures of *Microbacterium* LMB2 produce primarily AMPO after exposure to MBOA (Fig. S3B, 34).

To test community effects, we cultured the nonSC and metabolizing metSC in 96-well plates in a complex medium supplemented with 500 µM MBOA. We performed metabolite analyses to confirm their differential MBOA degradation and determine the various metabolization products at 68 h (Fig. S4, data need to be interpreted qualitatively, see note in MATERIALS AND METHODS). As expected, we found that the nonSC did not degrade, whereas the metSC degraded MBOA. However, the metSC formed HMPAA rather than AMPO (Fig. 1B). Over the course of the experiment, the levels of MBOA continuously decreased; HMPAA continuously increased; and not much AMPO accumulated (Fig. S4A). In addition, we also detected a metabolite feature with a mass corresponding to the intermediate AMP (acetylation of AMP yields HMPAA; Fig. S1) that accumulated over time similar to HMPAA (Fig. S4B). In summary, the two SynComs nonSC and metSC differ in their ability to metabolize MBOA. In contrast to pure cultures of LMB2 that primarily produce AMPO (Fig. S3B), we measured predominantly the MBOA metabolization product HMPAA in the metSC where LMB2 is present in combination with other strains. This finding indicates that *Microbacterium* LMB2 interacts with at least one other metSC strain, resulting in the formation of HMPAA.

### Most strains can form HMPAA together with LMB2

**Fig 2:**
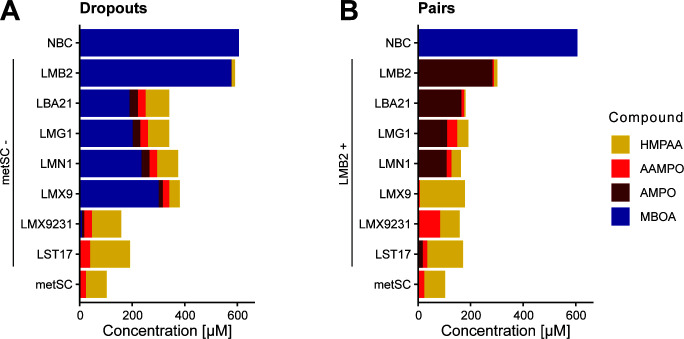
Metabolite profiles. MBOA, AMPO, AAMPO, and HMPAA were measured for dropout SynComs (**A**) and paired cultures (**B**) after growth with MBOA. Stacked bar graphs display the quantitative metabolite measurements. SynComs and paired strains were grown in 96-well plates in liquid 50% TSB supplemented with 500 µM of MBOA alongside their no bacteria controls (NBC, media with MBOA but without bacteria). Cultures were grown in triplicates and pooled before measurements (*n* = 1).

To identify the strain(s) that are involved in the HMPAA formation, we first tested reduced metSC communities in which individual strains were dropped out. We incubated these ‘dropout’ SynComs in a complex 50% TSB medium supplemented with 500 µM MBOA for 68 h and performed metabolite analyses to determine the major metabolization products (Fig. S1). First, the dropout SynCom without *Microbacterium* LMB2 did not markedly degrade MBOA (Fig. 2A), confirming the key role of LMB2 in initiating the metabolization of MBOA. Second, HMPAA was formed in all other dropout SynComs in which another strain was removed. While the dropout of *Enterobacter* LMX9231 and *Stenotrophomonas* LST17 did not markedly alter the metabolizing capacity of the metSC, considerable levels of MBOA were still detected in the dropout SynComs lacking any of the other four strains. Overall, this indicated that not one single specific strain is required for the formation of HMPAA in the metSC.

For further identification of individual strains interacting with *Microbacterium* LMB2 to form HMPAA, we tested each community member paired with LMB2 as in the assay above. Consistent with previous work (34; Fig. S3B), co-cultures of *Microbacterium* LMB2 with itself completely degraded MBOA and formed primarily AMPO (Fig. 2B). In co-cultures with *Bacillus* LBA21, *Streptomyces* LMG1, and *Chitinophaga* LMN1, AMPO remained as the primary product measured. In contrast, in co-cultures with *Pseudomonas* LMX9 and *Stenotrophomonas* LST17, mainly HMPAA was detected, similar to the full metSC. The co-culture with *Enterobacter* LMX9231 was special, where mainly HMPAA and AAMPO, the acetylated form of AMPO, were detected. Importantly, with the exception of the co-culture with *Bacillus* LBA21, most pairs formed some levels of HMPAA. The highest levels of HMPAA were detected in co-cultures with *Pseudomonas* LMX9 and *Stenotrophomonas* LST17, intermediate levels with *Enterobacter* LMX9231 and the full metSC, and low levels with *Streptomyces* LMG1 and *Chitinophaga* LMN1. Taken together, HMPAA can be formed in co-cultures of *Microbacterium* LMB2 with five out of the six core members of the metSC.

### Extracellular localization of AMP permits further metabolization by other strains

**Fig 3:**
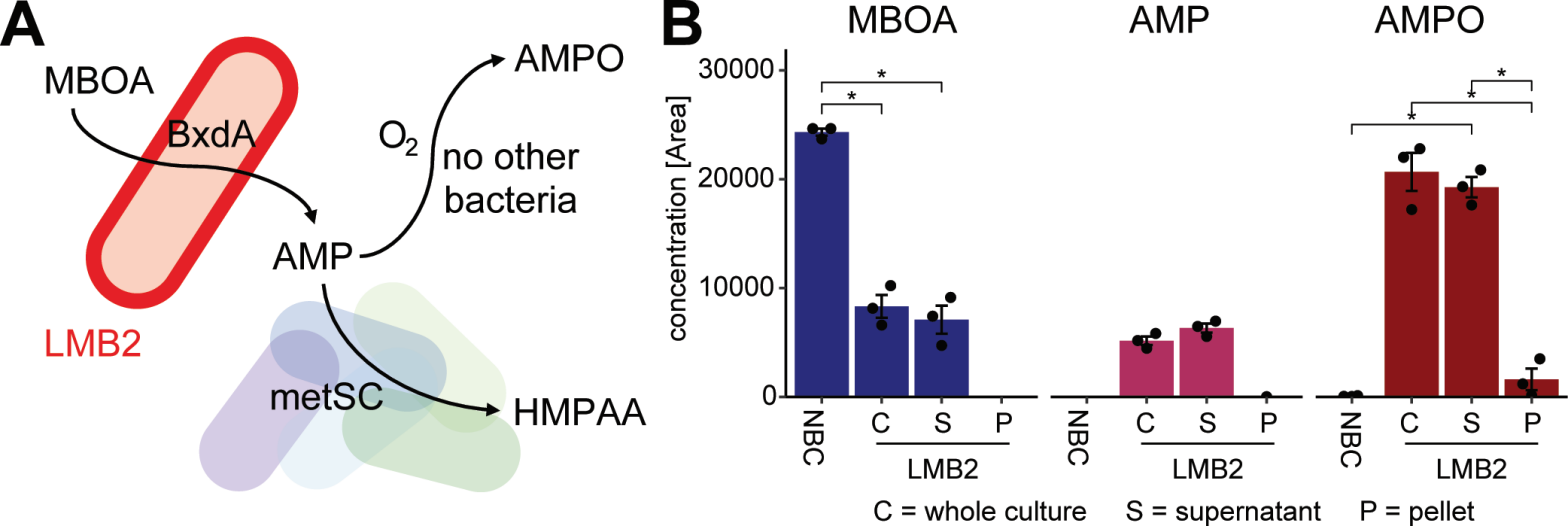
Model and localization of AMP. (**A**) Model for the degradation of MBOA in the absence of other bacteria or in the context of the metSC. The *Microbacterium* LMB2 initiates the degradation of MBOA by ring opening through the lactonase BxdA (predicted to be cytosolic), leading to the intermediate compound AMP. In the absence of other bacteria, AMP undergoes oxidative dimerization to AMPO, whereas five of the six strains of the metSC have the capacity to acetylate AMP to HMPAA. To become available to the other strains, the model proposes that AMP is localized extracellularly. (**B**) MBOA, AMP, and AMPO were measured in whole cultures (C), supernatant (S), and pellet (P) fractions of mono-cultures of LMB2. LMB2 was grown for 5 h in 96-well plates filled with 50% TSB medium supplemented with 500 µM of MBOA alongside their no bacteria controls (NBC, media with MBOA but without bacteria). Cultures were grown and measured in triplicates (*n* = 3). Asterisks indicate significant differences between comparisons (Welch’s *t*-test, Bonferroni-adjusted *P* < 0.05).

Based on chemistry (Fig. S1) and the above data (Fig. 2), we propose the following model (Fig. 3A): *Microbacterium* LMB2 initiates the metabolization of MBOA through BxdA (34), and the resulting intermediate AMP can then be further acetylated to HMPAA by five of the metSC strains. In the absence of these strains, the intermediate AMP undergoes oxidative dimerization to AMPO. This model implies that AMP is accessible to the other strains. BxdA is predicted to be a cytoplasmic protein (Fig. 3A, see methods), so either BxdA or AMP would need to be released from *Microbacterium* LMB2 cells for AMP to become available extracellularly. To test whether AMP is available extracellularly, we cultivated LMB2 in 50% TSB with 500 µM of MBOA for 5 h and measured metabolization products in whole culture, supernatant, and pellet fractions. By this time point, MBOA had been partially degraded, and both AMP and AMPO could be detected in whole culture and supernatant but not in the pellet fraction (Fig. 3B). Identifying AMP in the supernatant corroborates the proposed model: AMP localization outside of LMB2 cells permits further metabolization by the other strains of the metSC.

### The metabolizing SynCom exhibits growth in the presence of MBOA

**Fig 4:**
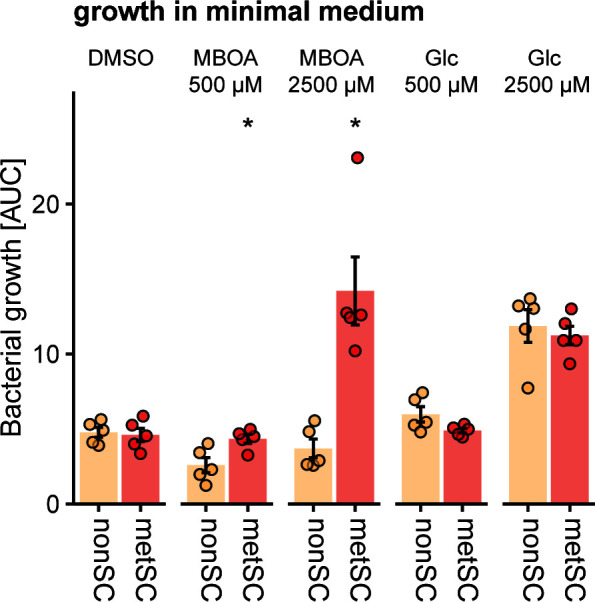
The metSC SynCom can use MBOA as the sole carbon source. The two SynComs were grown in 96-well plates in minimal medium supplemented with 500 and 2,500 µM MBOA or glucose (positive controls). Minimal medium supplemented with DMSO only was used as the negative control. Bacterial growth over 68 h is reported as the area under the growth curve (AUC) derived from OD_600_ measurements over time. Means ± standard error and individual data points are shown (*n* = 5). Asterisks indicate significant differences between treatment (pairwise *t*-test, Bonferroni-adjusted *P* < 0.05).

Next, we tested whether the ability to metabolize MBOA enables the metSC to use MBOA as a sole carbon source for bacterial growth. For this, we grew the nonSC and metSC SynComs in minimal media supplemented with 500 or 2,500 µM MBOA, glucose (Glc), or no additional carbon source (DMSO), keeping the DMSO concentration constant in all treatments. We quantified community growth by monitoring the optical density (OD_600_) over time. While both SynComs exhibited similar growth in the DMSO and glucose controls, the metSC grew significantly better in MBOA than the nonSC, particularly at the high concentration (Fig. 4). Testing all strains individually confirmed that *Microbacterium* LMB2 was the only strain capable of utilizing MBOA as a sole carbon source (Fig. S5). This analysis suggested that the metabolizing SynCom metSC benefitted from LMB2’s capacity to use MBOA as a sole carbon source. Of note, this conclusion comes with the caveat that the OD_600_ measurements do not discriminate between the growth of LMB2 and the other community members (see below).

### The capacity to metabolize MBOA affects community size and structure

**Fig 5:**
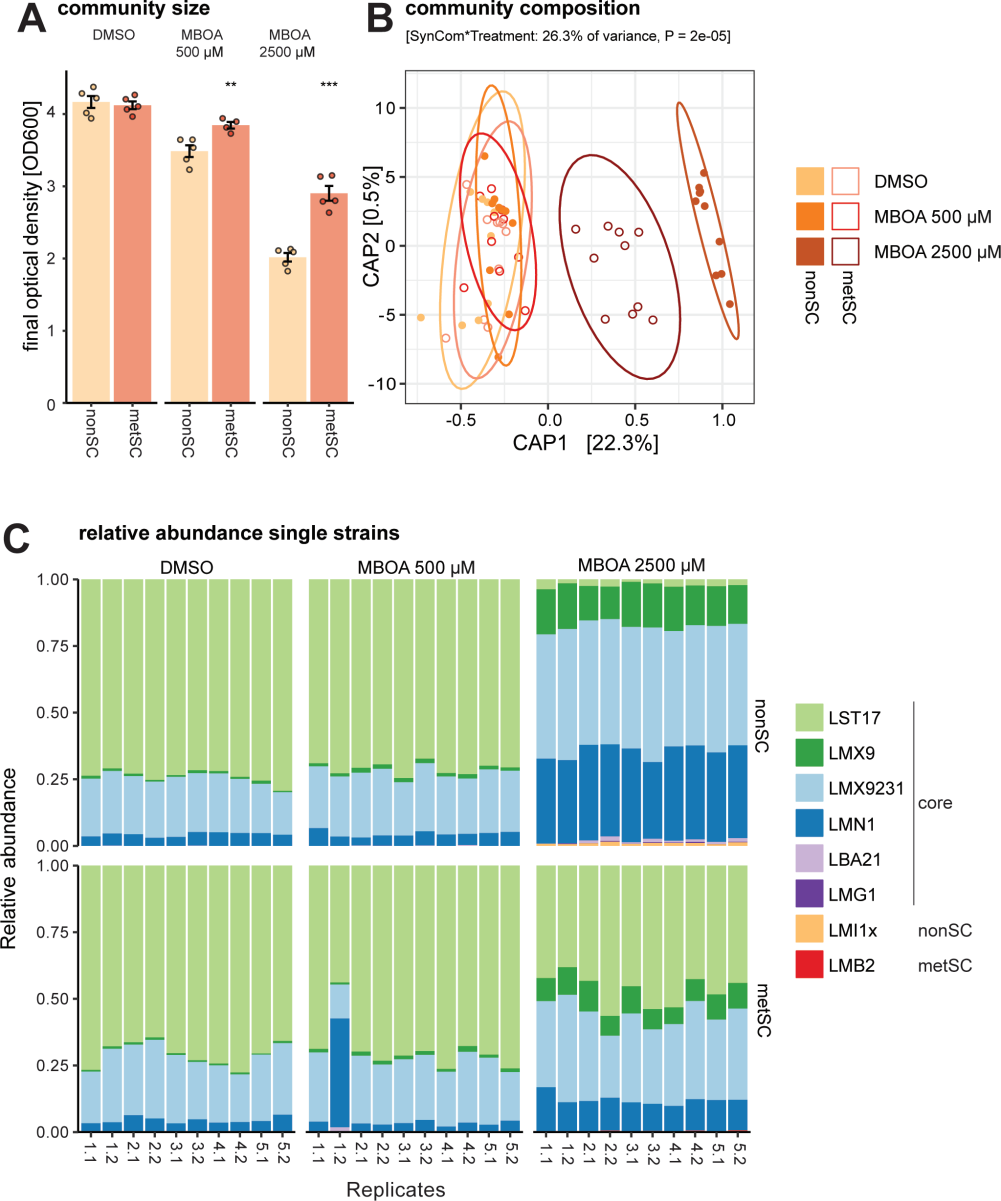
Size and composition of the non-SC and met-SC SynComs in the presence and absence of MBOA. SynComs were grown in shake flasks in 50% TSB supplemented with 500 or 2,500 µM MBOA or DMSO only for 68 h. Five replicate cultures were set up for each SynCom and treatment, and two parallel samples of each culture were collected for community analysis by 16S rRNA gene amplicon sequencing. (**A**) Bacterial growth determined by OD_600_ at the end of the experiment (*n* = 5). Asterisks indicate significant differences between the two SynComs (pairwise *t*-test, Bonferroni-adjusted *P* < 0.05). (**B** and **C**) Community compositions at the end of the experiment. Shown is a canonical analysis of principal coordinates (CAP) based on Bray-Curtis distances and the model ~SynCom * treatment (*n* = 10) (**B**) and relative abundances of the SynCom members for individual samples (**C**).

To examine the effect of the MBOA metabolization trait on community size, we next exposed both SynComs in 50% TSB medium to 500 or 2,500 µM of MBOA or a control treatment (DMSO) for 68 h. In addition to MBOA, this complex medium contains a mix of other carbon sources available to the microbes. For scale and sampling reasons, we performed this experiment in Erlenmeyer flasks and first confirmed that MBOA was degraded by the metSC but not by the nonSC in this experimental setting and that HMPAA is also the dominant degradation product under these conditions (Fig. S6A and B). We found that the total community size, as determined by the OD_600_ of the cultures, decreased with increasing levels of MBOA but that the metSC showed a larger community size than the nonSC in presence of MBOA (Fig. 5A). Complementarily, we also determined colony-forming units (CFU) of the cultures and found similar results (Fig. S6C), with both OD- and CFU-based community quantifications correlating well (Fig. S6D). This revealed that the ability to metabolize MBOA enabled the metSC community to reach a higher population size when faced with the antimicrobial compound.

In the same experiment, we also determined the community compositions of the two SynComs using amplicon sequencing. While the presence of MBOA significantly affected the community composition (PERMANOVA, *R*^2^ = 0.26, *P* = 0.001), no major effect was found for the two types of SynComs or their interaction with MBOA (Table S1). Constrained ordination visualized this MBOA effect along the first axis with SynCom compositions differing at the high MBOA concentration relative to communities at 500 µM MBOA and the DMSO controls (Fig. 5B). The high MBOA treatment (2,500 µM) had a stronger impact on the composition of the nonSC than on the metSC. In DMSO and at 500 µM of MBOA, both SynComs were compositionally dominated by *Stenotrophomonas* LST17, followed by lower levels of *Enterobacter* LMX9231 and *Chitinophaga* LMN1, while the other strains were either only detected at very low levels or not any more (Fig. 5C; Fig. S7A). Comparisons to the input samples collected at SynCom assembly confirmed that all strains were present, albeit gram-positive strains being low abundant in the initial SynComs (Fig. S7B). While the composition of the metSC was more similar compared to the control and 500 µM MBOA treatments, several core strains of both SynComs differed in their relative abundances at high MBOA levels: *Stenotrophomonas* LST17 (susceptible to MBOA; tolerance from Fig. 1A and Fig. S2), which made up about 70% of the community in the DMSO and MBOA 500 µM treatment, was strongly reduced to 22 and 47% in the nonSC and metSC, respectively. In contrast, *Enterobacter* LMX9231 (intermediate tolerance) and *Pseudomonas* LMX9 (tolerant), as well as *Chitinophaga* LMN1 (susceptible), increased in their relative abundances at high MBOA concentration relative to the DMSO and 500 µM treatments. In conclusion, MBOA reduces the bacterial community size (Fig. 5A) and influences the community composition more strongly if a SynCom is unable to metabolize the compound (Fig. 5B and C).

## DISCUSSION

Root-derived plant-specialized metabolites structure microbiomes (6) and select root-associated bacteria that can both tolerate (8) and/or metabolize (34) these compounds. In this study, we found that maize root bacteria assembled as a synthetic community interacted to metabolize benzoxazinoids *in vitro,* leading to the accumulation of the alternative MBOA-metabolization product, HMPAA. The capacity to metabolize benzoxazinoids was beneficial to the metSC SynCom, enhancing their tolerance to MBOA and enabling the community to reach higher population densities than the nonSC SynCom at high MBOA concentrations. Furthermore, the effect of MBOA on community composition was dependent on the metabolization phenotype. Below we discuss the underlying mechanisms and benefits for microbial communities.

### Interacting bacteria redirect BX metabolism

Members of microbial communities often interact to degrade complex chemicals, such as antimicrobial compounds, pollutants, and complex substrates, but also cooperate for substrate utilization (21). Here, we found that SynComs of maize root bacteria interacted in the metabolization of MBOA. Against the expectation from our previous study, where we reported that single-strain cultures of *Microbacterium* LMB2 metabolized MBOA to AMPO (34), we found that the MBOA metabolizing metSC SynCom formed HMPAA as the dominant metabolite. Since none of the metSC strains formed HMPAA in single-strain culture, we concluded that the HMPAA formation required the MBOA-degrading bacterium *Microbacterium* LMB2 in combination with at least one other strain. In paired cultures, *Pseudomonas* LMX9 had the strongest effect on MBOA degradation and led to complete AMPO absence; thus, this strain appeared to be the strongest contributor among the SynCom strains. A similar alternative product formation during benzoxazinoid degradation has previously been reported for a co-culture of the fungus *Fusarium verticillioides* with the bacterium *Bacillus mojavensis* (36). The *Fusarium* alone metabolized BOA to HPMA, but in co-culture with *Bacillus*, the intermediate AP was oxidized to APO. Since the *Fusarium* is susceptible to APO, while the *Bacillus* is not, this metabolic interaction resulted in inhibition of the pathogenic fungus without negatively affecting the bacterium. Here, we found that interacting bacteria redirected benzoxazinoid metabolization to an alternative compound. Future work is needed to validate if this redirected metabolization of MBOA to alternative HMPAA also occurs in the rhizosphere or in soil.

### Mechanism of alternative MBOA metabolization

The formation of the alternative MBOA metabolization product HMPAA by the metSC SynCom required at least two strains and was observed when *Microbacterium* LMB2 was present. Therefore, it is likely that LMB2 degraded MBOA by the previously identified lactonase BxdA (34) to the intermediate AMP, which is then acetylated by a second strain to HMPAA. This biochemical interaction model presents a localization challenge. Although BxdA is predicted to be a cytoplasmic protein, its reaction product, AMP, must become available outside of LMB2 cells to allow other strains to carry out the subsequent acetylation to HMPAA (Fig. 3A). While our results show that the majority of AMP is found extracellularly, further investigation is needed to determine whether BxdA is, at least in part, also present in the extracellular environment (despite *in silico* predictions) and/or whether MBOA and AMP are actively transported across the cell membrane. Parallel to solving this localization issue, identifying the enzyme(s) that acetylate AMP presents another research target.

For the latter, there is first evidence pointing toward the underlying biochemistry: *Pseudomonas chlororaphis*, for example, possesses an arylamine N-acetyltransferase (NAT1) that acetylates AP to HPAA (39). It is possible that some SynCom strains possess homologs of NAT1, which analogously could act on AMP, a methoxylated AP, resulting in the HMPAA formation. Further genomic and functional work with knockouts is required to confirm the role of NAT1 homologs in the HMPAA formation. Alternatively, a second pathway was proposed for the production of the acetamide HPAA: acylation of AP to the malonylated form HPMA followed by deacetylation to HPAA (33). Detoxification of benzoxazinoids via ring opening and malonylation is well known for fungal pathogens, such as *Fusarium* species (35, 40). Throughout our analyses, we did not detect the malonylated intermediate HMPMA. However, as HPMA can be degraded by microorganisms, such as bacilli with and without the accumulation of HPAA (33), the absence of HMPMA might also be explained by further degradation of such a product. Future work will focus on the identification and characterization of homologs of arylamine N-acyltransferases in the tested SynCom strains. *Pseudomonas* LMX9 will be especially interesting in this regard, as it is the only strain that led to the complete absence of AMPO in paired culture with *Microbacterium* LMB2 and also seemed to have the strongest effect on MBOA degradation in single dropouts of the metSC (Fig. 2). Our benzoxazinoid metabolizing SynCom, thus, offers a great toolbox to disentangle the biochemistry of bacterial interaction for the metabolization of plant-specialized metabolites.

### Interaction in benzoxazinoid metabolization is beneficial to community fitness

Benzoxazinoids were shown to selectively inhibit bacterial growth (8, 41), are metabolized by individual bacterial members of the maize root microbiome (34–36), and alter the composition of root microbial communities (11–14). Here, we found that the metSC SynCom was more tolerant to MBOA than the nonSC SynCom (Fig. 4A). MBOA altered not only the size but also the composition of both SynComs, implying that certain strains were more affected by the presence of MBOA than others (Fig. 4C). Specifically, high MBOA concentrations strongly reduced *Stenotrophomonas* LST17, which dominated the SynComs containing no or low amounts of MBOA, whereas *Pseudomonas* LMX9 and *Chitinophaga* LMN1 increased in relative abundance at the high MBOA concentrations. It is currently unclear whether this increase in LMX9 and LMN1 was due to an increase in absolute abundance or whether the abundance of the strains remained stable, and relative abundance solely increased due to the reduction of *Stenotrophomonas* LST17. For LST17, the inhibition was linked to its low tolerance to high concentrations of MBOA (Fig. S2). In the metSC SynCom, the *Stenotrophomonas* remained the most abundant strain, which could be explained by MBOA degradation and implies that this strain was also tolerant to the produced HMPAA. Having identified that HMPAA is formed in community context, it will be important to determine the toxicity and ecology of this compound in future work.

It is currently unclear how the population dynamics developed over the course of the experiment. Even though *Microbacterium* LMB2 is required for MBOA metabolization in the metSC, it was only detected at low concentrations at the end of the experiment. One possible explanation is due to technical bias, with gram-positive strains being underrepresented in DNA extraction and therefore being detected at low abundance already in the input communities (Fig. S7B) . Nevertheless, another possible explanation is that LMB2 was present at higher concentrations at the beginning of the experiment, but then after initiating the MBOA metabolization, it was overgrown by fast-growing and less MBOA-tolerant strains. In line with this, the fast-growing *Stenotrophomonas* LST17 (Fig. S2B) would have been first present at low abundance, and then would have started growing once the MBOA concentrations fell below inhibitory concentrations. Interestingly, *Chitinophaga* LMN1, which also showed hardly any growth at 2,500 µM MBOA, increased stronger in relative abundance in the nonSC than in the metSC at high MBOA. This suggested that factors other than MBOA tolerance contributed to the final SynCom composition, such as microbe-microbe interactions based on metabolic exchanges between SynCom strains or modulation of direct competitive interactions (42, 43). Cross-feeding was, for example, previously identified in a seven-member community of maize root bacteria (44). An *Enterobacter* had been identified as keystone species in this SynCom (45). The keystone *Enterobacter* had the broadest substrate utilization of maize root extracts, and its dropout decreased carbohydrate utilization of the SynCom (44). Our SynCom also contained an *Enterobacter* showing that high abundance and substrate use may further explain community structure independent of tolerance to benzoxazinoids. Nevertheless, our observation that benzoxazinoids structured SynComs also *in vitro* underlines the importance of benzoxazinoids in shaping root-associated microbial communities on benzoxazinoid-producing maize roots (11–14).

### Effect of benzoxazinoid metabolization for bacterial growth and potential effects on host health

The metabolization of benzoxazinoids by microbes could serve different purposes: whether it is to detoxify them, profit from a carbon source, use degradation products as defense against other microbiota members, or whether degradation is beneficial for the host. We found that a benzoxazinoid-metabolizing SynCom was more tolerant to MBOA at high MBOA concentrations, which suggests specific adaptation of host microbiomes to tolerate and metabolize host-specialized metabolites and potentially presents a strategy for rhizosphere bacteria to access an extra carbon source to thrive in the rhizosphere (44, 46). Indeed, MBOA can be used as a sole carbon source by the degrading *Microbacterium* LMB2 (34). However, our SynCom experiments in complex medium suggest that, under the tested conditions, MBOA metabolization served mainly for detoxification for some microbiome members rather than strongly contributing to the overall population size as a separate carbon source. As the degradation of specialized metabolites can be highly context and environment-dependent, the SynCom work could be next transferred to a gnotobiotic plant system as tested by Niu et al. (45). Members of the genus *Stenotrophomonas* show beneficial effects for plant growth and health (47). In a first step, one could, therefore, focus on whether the presence of MBOA metabolization increases local *Stenotrophomonas* abundance also *in planta* and potentially affects SynCom functions supplied to the host, such as protection against plant pathogens. Alternatively, benzoxazinoid degradation and degradation products could additionally influence community function as regulators (48). Given the widespread nature of microbial interactions and the diversity of plant-specialized metabolites, we propose that microbial interactions affect how microbial communities cope with plant specialized metabolites across the plant kingdom and thereby also potentially influence plant fitness.

### Conclusion

Natural microbial communities are diverse, and single members interact to fulfill a complex task or use resources efficiently (21). Here, we report that maize root bacteria interact to tolerate and metabolize benzoxazinoids. Especially at high benzoxazinoid concentrations, the interaction of maize root bacteria positively affected the performance as a community. Our findings highlight the importance of studying plant-specialized metabolites mediating chemical communication in plant-microbiome interactions in more complex synthetic or natural microbial communities.

## MATERIALS AND METHODS

### Bacteria, SynCom assembly, and experimental setup

Maize root bacteria (i.e., MRB collection, 8) were routinely grown on solid 100% TSA plates (30 g/L tryptic soy broth and 15 g/L of agar, both Sigma-Aldrich) or in liquid 50% TSB medium (15 g/L tryptic soy broth) with 180 rpm shaking at 25–28°C. For cryo stocks, bacteria were grown for 48 h in liquid 100% TSB (30 g/L tryptic soy broth) and mixed with sterile-filtered glycerol (Sigma-Aldrich) at a final concentration of 20%.

The rationales for SynCom assembly are detailed in the Supplementary methods. In brief, the SynComs consisted of the six core strains *Stenotrophomonas* LST17, *Pseudomonas* LMX9, *Enterobacter* LMX9231, *Chitinophaga* LMN1, *Bacillus* LBA21, and *Streptomyces* LMG1. With the seventh strain, the SynComs differed in their ability to metabolize MBOA: *Microbacterium* LMI1x was unable to metabolize MBOA and was selected for the *non*-metabolizing SynCom (nonSC). *Microbacterium* LMB2 possessed the lactonase BxdA permitting to metabolize MBOA to AMPO and was part of the MBOA-*metabolizing* SynCom (metSC). BxdA was predicted to be a cytoplasmic protein (SignalP 6.0 does not identify any known secretory signal peptide, 49).

The individual strains were pre-grown in liquid cultures for the different experiments. For experiments depicted in Fig. 1, 2 and 4, pre-cultures were inoculated from single colonies and grown in 1 mL of liquid 50% TSB in 2 mL 96-well deep-well plates (Semadeni, Ostermundigen, Switzerland). Pre-culture plates were covered with a Breathe-Easy membrane (Diversified Biotech, Dedham, USA) and grown until stationary phase for 4 days at 28°C and 180 rpm.

For the preparation of SynComs or strain mixes, cultures of individual strains were adjusted to OD_600_ = 0.6 and the required strains mixed at equal ratios. Main cultures were inoculated by adding 4 µL of the adjusted mixes/single strains into 96-well microtiter plates (Corning, Corning, USA) containing 200 µL of fresh liquid media, including the compounds at the concentrations to be tested. The chemical treatments were prepared at the desired concentrations by mixing their stock solutions into the liquid growth media. The stock solution for MBOA (Sigma-Aldrich) was prepared in the solvent DMSO (Sigma-Aldrich) at 606 mM (100 mg/mL). MBOA was added to the medium at final concentrations of 0, 500, or 2,500 µM, and the DMSO concentration was kept constant in all treatments, including the controls.

We mainly utilized our previously described 96-well liquid culture-based growth system (8). In brief, bacterial growth was monitored in 96-well plates in a high-throughput manner using a stacker (BioStack 4, Agilent Technologies, Santa Clara, USA), which was connected to a plate reader (Synergy H1, Agilent Technologies, Santa Clara, USA). Briefly, the 96-well plates were incubated at ambient temperature, and each plate was transferred to the plate reader approximately every 95 min (see Supplementary methods for details). Within the plate reader, the cultures were shaken for 2 min (linear shaking at 567 cpm) before the OD_600_ readings were recorded. In each plate, wells with growth medium were included as no bacteria controls (NBC), and in each run, one plate containing only media was included to monitor potential contaminations. For assessing the community structure, precultures and SynComs were grown in Erlenmeyer flasks instead of 96-well plates, and growth was assessed by OD_600_ measurements using a biophotometer (Eppendorf, Hamburg, Germany) and by dilution series plating and colony-forming unit determination.

For details on the different experiments performed, see Supplementary methods.

### Data analysis

We used the R version 4.4 (R core Team, 2024) for all statistical analyses and visualization of the data. We first inspected the bacterial growth data for normality using Shapiro-Wilk tests. Differences between the two SynComs (nonSC vs. metSC) and treatments were assessed using pairwise *t*-tests and reported for the different treatments in the graphs. *P*-values were adjusted for multiple hypothesis testing using the Bonferroni method within R. We used the following packages for data analysis and visualizations: Tidyverse (50), Broom (51), DECIPHER (52), DESeq2 (53), emmeans (54), ggthemes (55), pheatmap (56), multcomp (57), phyloseq (58), phytools (59), and vegan (60) in combination with custom functions. All source data and R code used for statistical analysis and graphing are available from GitHub.

### Metabolite analysis from bacterial cultures

Samples for metabolite analyses were processed and measured as described previously (34). Briefly, metabolites in bacterial cultures were fixed in 70% MeOH + 0.1% FA. Replicates of the same sample group were pooled and diluted, followed by filtration and centrifugation to remove bacterial debris. Benzoxazinoids and their degradation products were profiled using an Acquity I-Class UHPLC System (Waters, Milford, USA) coupled to a Xevo G2-XS QTOF mass spectrometer (Waters, Milford, USA) equipped with a LockSpray dual electrospray ion source (Waters, Milford, USA). Different benzoxazinoid standard compounds were run together with the samples at varying concentrations and used for quantification and identification. In this work, we only report MBOA, AMPO, AAMPO, and HMPAA. For details, see Supplementary methods. All source data and R code used for graphing are available from GitHub.

*Note for the interpretation of the data:* Although we quantified most compounds in most experiments with analytical standards, the data should be considered as semi-quantitative or rather interpreted qualitatively. For instance, conclusions on C balance or stoichiometry between compounds cannot be drawn. Reasons are manyfold: (i) the data do not reflect accumulated endpoint measures, but snapshots of a given timepoint of compounds that can be under continued metabolization by some of the tested bacteria; (ii) diverse bacteria may metabolize the compounds to (further) yet unknown and unaccounted degradation products; (iii) AMP (also its analytical standard) is unstable (a bit of O_2_ is sufficient to transform AMP to AMPO); and (iv) AMPO is underestimated because it is highly hydrophobic; therefore, it is prone to precipitate and losses due to being sticky to vessels (losses due to pipette tips or filters). Only data interpretation within an analytical run and relative across treatments within a compound is meaningful.

### Community analysis

DNA was extracted from 1 mL of SynCom samples (Experiment 7) using the NucleoSpin Soil kit (Macherey-Nagel, Düren, Germany) following the manufacturer’s instructions (see also Supplementary Methods). The 16S rRNA gene was amplified in a two-step PCR, with custom barcodes for sample identification added in the second PCR. The detailed methods for PCR amplification and their primers, product purification, and pooling are described in the Supplementary methods. The pooled library was sequenced on a MiSeq instrument (Illumina, Inc., San Diego, USA) with a v2 nano flow cell and the 2 × 250 bp pair-end sequencing protocol at the Next-Generation Sequencing Platform (https://www.ngs.unibe.ch, University of Bern, Switzerland). The raw sequencing data were deposited at the European Nucleotide Archive (http://www.ebi.ac.uk/ena) under project number PRJEB86484.

The raw sequencing data were processed in R using the DADA2 pipeline version 1.20 (61). For details, see Supplementary Methods. The 16S rRNA gene sequences of the SynCom members were mapped to the amplicon sequences using usearch (62) with an identity of 0.97. The counts of the mapped ASVs were normalized by rarefaction using phyloseq (58), and the abundances of the strains were further adjusted based on their estimated copy numbers of the 16S rRNA genes (Dataset S1). Compositional differences between the SynComs and by the treatments were assessed using permutational analysis of variance (PERMANOVA, 99,999 permutations; model: ~SynCom * Treatment) on Bray-Curtis distances using the R package vegan (60). The effects on community composition using the same model were visualized with a canonical analysis of principal coordinates (CAP) using the R package phyloseq (58). All source data and code used for statistical analysis and graphing are available from GitHub.

## Data Availability

All source data are made publicly available. The sequencing data are available from the European Nucleotide Archive as Bioproject PRJEB86484. We provide all data on bacterial growth and metabolite data together with their analysis code on GitHub. All codes used for statistical analysis and graphing are available from GitHub.

## SUPPLEMENTAL MATERIAL

Data set S1 msphere.00159-25-s0001.xlsx Information on all bacterial strains used in the SynCom, taxonomy information, 16S Sanger sequence, and number of 16S copies.

Data set S2 msphere.00159-25-s0002.html Documentation of the DADA2 pipeline script used.

Supplemental Information msphere.00159-25-s0003.pdf Supplemental methods, results, references, figures, and table.

ASM does not own the copyrights to Supplemental Material that may be linked to, or accessed through, an article. The authors have granted ASM a non-exclusive, world-wide license to publish the Supplemental Material files. Please contact the corresponding author directly for reuse.
