# Supplementary material for: Synthetic communities of maize root bacteria interact and redirect benzoxazinoid metabolization
Source: mSphere. 2025 Aug 25;10(9):e00159-25. doi: 10.1128/msphere.00159-25 (PMC12483121; doi:10.1128/msphere.00159-25)
Supplement: Data set S2 — Documentation of the DADA2 pipeline script used. [file msphere.00159-25-s0002.html]

Syncom: Initial analysis


# Syncom: Initial analysis

#### Marco Kreuzer

Interfaculty Bioinformatics Unit (IBU)  
marco.kreuzer@bioinformatics.unibe.ch

#### 10.09.2021

## 0.1 Workflow

The dada2 pipeline was applied in order to get high-quality 16S sequences
from raw reads.

The pipeline consists of the following steps:

1. filtering and trimming of raw reads

```
filterAndTrim(...,
              maxN=0, 
              maxEE=c(2,2), 
              trimLeft = 19,
              trimRight = 19,
              truncQ=2, 
              rm.phix=TRUE,
        compress=TRUE, multithread=FALSE)
```

2. Learning errors from the quality filtered reads

```
errF <- learnErrors(..., multithread=TRUE)
errR <- learnErrors(..., multithread=TRUE)
```

The estimations of the error rates looks as expected:

3. The filtered sequences were dereplicated:

```
derepFs <- derepFastq(..., verbose=TRUE)
```

4. The dada2 algorithm for denoising the reads was applied

```
 dada(derepFs, err=errF, multithread=TRUE)
```

5. Then the reads pairs were merged

```
 mergers <- mergePairs(dadaFs, derepFs, dadaRs, derepRs, verbose=TRUE,
                      minOverlap=20)
```

6. Chimeric sequences were removed as follows:

```
seqtab.nochim <- removeBimeraDenovo(..., 
                                    method="consensus", 
                                    multithread=TRUE, verbose=TRUE)
```

7. The taxonomy was assigned in two searches against the SILVA database.

Using the “silva\_nr\_v138\_train\_set.fa.gz” data for the assignment until genus
level.

```
assignTaxonomy(..., "silva_nr_v138_train_set.fa.gz",
                       multithread=TRUE)
```

Using the “silva\_species\_assignment\_v138.fa.gz” data for the assignment until genus
level.

```
addSpecies((..., "silva_species_assignment_v138.fa.gz")
```

8. A phyloseq object with the taxonomy and the counts tables was built.

## 0.2 QC

Boxplot of the mean sequence length per sample before and after merging. This is an important validation that the filtering and merging parameters were set correctly.

### 0.2.1 High-level taxonomic comparison between controls and tissue samples

As can be seen in the plot below, only Bacterial reads were amplified.

Figure 1: Only Bacterial reads were sequenced

### 0.2.2 Read Track over all barcode combinations sequenced

| Sample | number\_of\_reads |
| --- | --- |
| BE11\_1 | 7806 |
| BE11\_2 | 5673 |
| BE11\_3 | 6949 |
| BE11\_4 | 7459 |
| BE11\_5 | 6472 |
| BE11\_6 | 6455 |
| BE11\_7 | 8148 |
| BE11\_8 | 6080 |
| BE11\_9 | 8288 |
| BE11\_10 | 10229 |
| BE11\_11 | 6468 |
| BE11\_12 | 6004 |
| BE11\_13 | 7845 |
| BE11\_14 | 7114 |
| BE11\_15 | 7431 |
| BE11\_16 | 5624 |
| BE11\_17 | 4584 |
| BE11\_18 | 3111 |
| BE11\_19 | 2462 |
| BE11\_20 | 7250 |
| BE11\_21 | 3442 |
| BE11\_22 | 1383 |
| BE11\_23 | 7554 |
| BE11\_24 | 6623 |
| BE11\_25 | 5019 |
| BE11\_26 | 2866 |
| BE11\_27 | 3183 |
| BE11\_28 | 3029 |
| BE11\_29 | 3173 |
| BE11\_30 | 3963 |
| BE11\_31 | 8967 |
| BE11\_32 | 3642 |
| BE11\_33 | 4396 |
| BE11\_34 | 3816 |
| BE11\_35 | 3218 |
| BE11\_36 | 3500 |
| BE11\_37 | 5770 |
| BE11\_38 | 8891 |
| BE11\_39 | 7205 |
| BE11\_40 | 9868 |
| BE11\_41 | 4852 |
| BE11\_42 | 3093 |
| BE11\_43 | 2829 |
| BE11\_44 | 5687 |
| BE11\_45 | 5361 |
| BE11\_46 | 7778 |
| BE11\_47 | 6921 |
| BE11\_48 | 6743 |
| BE11\_49 | 4440 |
| BE11\_50 | 3381 |
| BE11\_51 | 3227 |
| BE11\_52 | 6206 |
| BE11\_53 | 6872 |
| BE11\_54 | 3794 |
| BE11\_55 | 7125 |
| BE11\_56 | 4413 |
| BE11\_57 | 5307 |
| BE11\_58 | 9125 |
| BE11\_59 | 7774 |
| BE11\_60 | 7753 |
| BE11\_61 | 8165 |
| BE11\_62 | 9455 |
| BE11\_63 | 6621 |
| BE11\_64 | 9120 |
| BE11\_65 | 5904 |
| BE11\_66 | 2750 |
| BE11\_67 | 7505 |
| BE11\_68 | 5460 |
| BE11\_69 | 3372 |
| BE11\_70 | 8624 |
| BE11\_71 | 7050 |
| BE11\_72 | 4895 |
| BE11\_73 | 6715 |
| BE11\_74 | 6945 |
| BE11\_75 | 3226 |
| BE11\_76 | 11020 |
| BE11\_77 | 8509 |
| BE11\_78 | 5454 |
| BE11\_79 | 3850 |
| BE11\_80 | 7047 |
| BE11\_81 | 5623 |
| BE11\_82 | 6620 |
| BE11\_83 | 6228 |
| BE11\_84 | 7422 |
| BE11\_85 | 6891 |
| BE11\_86 | 6994 |
| BE11\_87 | 12702 |
| BE11\_88 | 7367 |
| BE11\_89 | 6920 |
| BE11\_90 | 7810 |
| BE11\_91 | 54 |
| BE11\_92 | 55 |
| BE11\_93 | 18 |
| BE11\_94 | 6832 |
| BE11\_95 | 9 |
| BE11\_96 | 45294 |
| BE11\_97 | 1 |
| BE11\_99 | 4 |
| BE11\_101 | 2 |
| BE11\_102 | 12 |
| BE11\_103 | 2 |
| BE11\_105 | 2 |
| BE11\_106 | 1 |
| BE11\_107 | 1 |
| BE11\_109 | 6 |
| BE11\_110 | 1 |
| BE11\_112 | 10 |
| BE11\_114 | 4 |
| BE11\_115 | 3 |
| BE11\_116 | 3 |
| BE11\_117 | 3 |
| BE11\_118 | 1 |
| BE11\_119 | 3 |
| BE11\_120 | 2 |
| BE11\_121 | 3 |
| BE11\_122 | 4 |
| BE11\_123 | 2 |
| BE11\_125 | 3 |
| BE11\_127 | 3 |
| BE11\_128 | 5 |
| BE11\_130 | 1 |
| BE11\_131 | 2 |
| BE11\_132 | 2 |
| BE11\_133 | 26 |
| BE11\_134 | 2 |
| BE11\_135 | 1 |
| BE11\_136 | 6 |
| BE11\_137 | 1 |
| BE11\_139 | 5 |
| BE11\_142 | 6 |
| BE11\_145 | 1 |
| BE11\_146 | 1 |
| BE11\_147 | 1 |
| BE11\_149 | 2 |
| BE11\_150 | 1 |
| BE11\_151 | 1 |
| BE11\_152 | 2 |
| BE11\_155 | 2 |
| BE11\_156 | 1 |
| BE11\_157 | 3 |
| BE11\_158 | 1 |
| BE11\_159 | 2 |
| BE11\_160 | 1 |
| BE11\_161 | 1 |
| BE11\_162 | 2 |
| BE11\_163 | 1 |
| BE11\_164 | 7 |
| BE11\_165 | 1 |
| BE11\_166 | 3 |
| BE11\_167 | 3 |
| BE11\_169 | 1 |
| BE11\_170 | 3 |
| BE11\_171 | 1 |
| BE11\_173 | 8 |
| BE11\_175 | 1 |
| BE11\_177 | 1 |
| BE11\_178 | 2 |
| BE11\_179 | 1 |
| BE11\_181 | 4 |
| BE11\_184 | 1 |
| BE11\_189 | 11 |
| BE11\_190 | 2 |
| BE11\_193 | 6 |
| BE11\_194 | 9 |
| BE11\_196 | 1 |
| BE11\_197 | 3 |
| BE11\_199 | 8 |
| BE11\_200 | 7 |
| BE11\_201 | 4 |
| BE11\_203 | 7 |
| BE11\_205 | 6 |
| BE11\_206 | 25 |
| BE11\_207 | 4 |
| BE11\_208 | 3 |
| BE11\_209 | 6 |
| BE11\_211 | 4 |
| BE11\_212 | 4 |
| BE11\_213 | 6 |
| BE11\_214 | 1 |
| BE11\_215 | 9 |
| BE11\_216 | 7 |
| BE11\_217 | 1 |
| BE11\_218 | 3 |
| BE11\_221 | 5 |
| BE11\_222 | 1 |
| BE11\_223 | 2 |
| BE11\_224 | 2 |
| BE11\_227 | 12 |
| BE11\_228 | 1 |
| BE11\_229 | 38 |
| BE11\_230 | 1 |
| BE11\_231 | 1 |
| BE11\_232 | 1 |
| BE11\_233 | 3 |
| BE11\_234 | 9 |
| BE11\_238 | 3 |
| BE11\_239 | 1 |
| BE11\_240 | 1 |
| BE11\_241 | 1 |
| BE11\_242 | 4 |
| BE11\_243 | 4 |
| BE11\_244 | 15 |
| BE11\_246 | 1 |
| BE11\_247 | 9 |
| BE11\_248 | 1 |
| BE11\_249 | 5 |
| BE11\_250 | 3 |
| BE11\_251 | 2 |
| BE11\_252 | 3 |
| BE11\_253 | 1 |
| BE11\_254 | 2 |
| BE11\_255 | 2 |
| BE11\_256 | 3 |
| BE11\_257 | 15 |
| BE11\_258 | 7 |
| BE11\_260 | 2 |
| BE11\_261 | 9 |
| BE11\_262 | 1 |
| BE11\_264 | 21 |
| BE11\_265 | 1 |
| BE11\_266 | 4 |
| BE11\_267 | 2 |
| BE11\_268 | 3 |
| BE11\_269 | 4 |
| BE11\_271 | 4 |
| BE11\_272 | 14 |
| BE11\_273 | 1 |
| BE11\_274 | 4 |
| BE11\_276 | 4 |
| BE11\_277 | 1 |
| BE11\_279 | 5 |
| BE11\_280 | 9 |
| BE11\_281 | 1 |
| BE11\_282 | 4 |
| BE11\_283 | 1 |
| BE11\_284 | 5 |
| BE11\_285 | 11 |
| BE11\_286 | 4 |
| BE11\_287 | 2 |
| BE11\_288 | 15 |
| BE11\_289 | 1 |
| BE11\_290 | 3 |
| BE11\_292 | 1 |
| BE11\_293 | 2 |
| BE11\_295 | 3 |
| BE11\_296 | 19 |
| BE11\_299 | 1 |
| BE11\_300 | 1 |
| BE11\_302 | 30 |
| BE11\_303 | 2 |
| BE11\_304 | 2 |
| BE11\_305 | 3 |
| BE11\_306 | 3 |
| BE11\_307 | 1 |
| BE11\_308 | 15 |
| BE11\_310 | 1 |
| BE11\_311 | 1 |
| BE11\_312 | 8 |
| BE11\_314 | 10 |
| BE11\_315 | 2 |
| BE11\_316 | 2 |
| BE11\_321 | 5 |
| BE11\_324 | 2 |
| BE11\_325 | 2 |
| BE11\_327 | 2 |
| BE11\_328 | 2 |
| BE11\_329 | 1 |
| BE11\_332 | 3 |
| BE11\_334 | 2 |
| BE11\_337 | 2 |
| BE11\_338 | 5 |
| BE11\_341 | 13 |
| BE11\_344 | 2 |
| BE11\_345 | 3 |
| BE11\_348 | 1 |
| BE11\_350 | 2 |
| BE11\_355 | 1 |
| BE11\_357 | 1 |
| BE11\_358 | 3 |
| BE11\_359 | 2 |
| BE11\_361 | 2 |
| BE11\_362 | 1 |
| BE11\_364 | 3 |
| BE11\_370 | 1 |
| BE11\_371 | 1 |
| BE11\_372 | 1 |
| BE11\_374 | 1 |
| BE11\_375 | 1 |
| BE11\_376 | 1 |
| BE11\_377 | 1 |
| BE11\_378 | 1 |
| BE11\_379 | 1 |
| BE11\_384 | 1 |

## 0.3 Genus-level assignments

The plot below shows the taxonomic assignment on the genus level (no species-level assignments were successfully made).

Figure 2: Genus-level assignments
