# Supplementary material for: Synthetic communities of maize root bacteria interact and redirect benzoxazinoid metabolization
Source: mSphere. 2025 Aug 25;10(9):e00159-25. doi: 10.1128/msphere.00159-25 (PMC12483121; doi:10.1128/msphere.00159-25)
Supplement: Supplemental Information — Supplemental methods, results, references, figures, and table. [file msphere.00159-25-s0003.pdf]

Supplementary Information

Synthetic communities of maize root bacteria interact and redirect benzoxazinoid metabolization

Lisa Thoenen[1,2], Christine Pestalozzi[2], Tobias Zuest[1,3], Marco Kreuzer[4], Pierre Mateo[1], Mikiko Karasawa[2], Gabriel Deslandes[1], Christelle A.M. Robert[1], Rémy Bruggmann[4], Matthias Erb[1], Klaus Schlaeppi[1,2 *]

[1] Institute of Plant Sciences, University of Bern, Bern, Switzerland
[2] Department of Environmental Sciences, University of Basel, Basel, Switzerland
[3] Department of Systematic and Evolutionary Botany, University of Zurich, Zurich, Switzerland
[4] Interfaculty Bioinformatics Unit, University of Bern, Bern, Switzerland
* Corresponding author: Klaus Schlaeppi, klaus.schlaeppi@unibas.ch

Running Head: "Benzoxazinoid metabolization in synthetic communities" (49 ch.)

Index

- Supplementary Methods
- Supplementary Results
- Supplementary Figures
- Supplementary Tables
- Supplementary References

Supplementary Methods

SynCom Design

For the synthetic communities tested here, we selected strains from the maize root bacteria (MRB collection, (1)). All strains were isolated from roots of soil-grown wild-type B73 maize plants. We have characterized most MRB strains for their tolerance to a range of benzoxazinoids (1) as well for their ability to metabolize MBOA in pure culture (2). In this study, we investigated the MBOA metabolization in a community context, with a particular focus on how this process influences the abundance of MBOA-sensitive strains. We designed two communities – the *non*-metabolizing SynCom, nonSC, and an MBOA-*metabolizing* SynCom, metSC – that differed in their ability to degrade MBOA. To this end, we included a strain capable of degrading MBOA in the metSC, while a closely related strain lacking this capability was added to the nonSC. We knew that strains of the Microbacteriaceae were phenotypically heterogenous with regards to MBOA metabolization (2), Therefore, we selected the well-characterized *Microbacterium* LMB2 as a MBOA degrader (2) for the metSC and the non-degrader *Microbacterium* LMI1x for the nonSC due to its MBOA tolerance (1). LMB2 and LMI1x are phylogenetically closely related based on their 16S rRNA gene sequence (1).

For the rest of the community, we filtered the MRB strains for absence of MBOA degradation in liquid culture (this excluded the Sphingomonadaceae and Rhizobiaceae). We then selected strains, one each from the major families of the MRB collection (branches of the phylogenetic tree, (1,2)). This resulted in 6 taxonomically distinct strains that build the core of both SynComs. We wanted to have representatives of Proteobacteriota, Actinobacteriota and Bacillota and that they have different levels of tolerance to MBOA. We chose genera that are often found in association with plant roots and selected strains of the following genera *Stenotrophomonas* (strain LST17), *Pseudomonas* (LMX9), *Enterobacter* (LMX9231), *Chitinophaga* (LMN1), *Bacillus* (LBA21) and *Streptomyces* (LMG1). Finally, we assured that the core strains and also the two Microbacteria LMB2 and LMI1x were distinguishable by short read 16S rRNA gene amplicon sequencing (i.e. strains were represented by different ASVs on the gene fragment amplified with the primers 515-F and 806-R).

Experiments

Experiment 1 (Figure 1A and S2): To determine the growth of the individual SynCom members in presence of MBOA, we cultured 5 replicates of each strain in 96-well plates containing liquid 50% TSB supplemented with MBOA (500 and 2'500 µM) or DMSO (negative control). We incubated the 96-well plates at ambient temperature (24-26°C), recorded the OD_{600} of the cultures roughly every 95 min and stopped the experiment at 68 h. For a measure of bacterial

growth, we calculated the area under the growth curve (AUC, x-axis for time and y-axis for OD_{600}) of the OD_{600} readings using the function *auc()* from package MESS (3) in R. We normalized growth in each treatment relative to the control. See analysis scripts for details.

Experiment 2 (Figure 1B and S4): We grew the nonSC and metSC SynComs in liquid 50% TSB supplemented with 500 µM MBOA in a time-series to characterize the kinetics of MBOA degradation and AMPO or HMPAA formation. Plates were incubated at ambient temperature (24-26°C) and shaken for 2 min (linear, 567 cpm) roughly every 95 min. For metabolite sampling, we removed replicate plates from the stacker at 16, 24, 44, 68 and 96 h. SynCom and NBC samples were grown in triplicates and pooled 1:1:1 for metabolite analysis. Fixing of bacterial cultures and metabolite analysis was performed as detailed below.

Experiment 3 (Figure S3): We specifically validated the weak capacity of *Enterobacter* LMX9231 to degrade MBOA relative to *Microbacterium* LMB2 in 96-well plates (26-28°C, 2 min of linear shaking at 567 cpm roughly every 15 mins) and in shake flasks (28°C, continuous shaking with 180 rpm). We grew the strains in triplicates in liquid 50% TSB supplemented with 500 µM MBOA or DMSO (negative control). Shake flask cultures (20 mL in 100 mL shake flask) were inoculated from triplicate overnight pre-cultures (50% TSB) at a starting OD of 0.2. Also "no bacteria controls" (NBC) with medium without bacteria were prepared. The experiments were stopped at 68-70 h and samples for metabolite measurements were fixed as described below. Metabolite measurements were performed on individual cultures (n = 3) or pools of three replicate DMSO controls (n = 1).

Experiment 4 (Figure 2): We tested relative to the metSC, single strain dropout SynComs (the metSC, minus the indicated strain) and each strain in paired cultures with *Microbacterium* LMB2. These assays were performed in liquid 50% TSB containing 500 µM MBOA. Plates were incubated at ambient temperature (24-26°C) and shaken for 2 min (linear, 567 cpm) roughly every 95 min. Assays were stopped at 68 h. SynComs, the paired cultures and their "no bacteria controls" (NBC, media with MBOA but without bacteria) were grown in triplicates and pooled 1:1:1 for metabolite analysis. Fixing of bacterial cultures and metabolite analysis was performed as detailed below.

Experiment 5 (Figure 3): To assess whether AMP is available outside of cells, we cultured LMB2 in triplicates as described for Experiment 3. Also "no bacteria controls" (NBC) with medium without bacteria were prepared. After 5 h of cultivation, we took samples for metabolite measurements in whole cultures as well as in supernatant and pellet fractions to test for AMP localization. Supernatant and pellet were recovered from 1 mL cultures of LMB2 that were pelleted at 3200x g for 10 min. The supernatant was transferred to a fresh tube and the pellet was washed once with fresh 50% TSB and resuspended in 1 mL of fresh 50% TSB for metabolite

analysis. Metabolite samples were fixed and analyzed as detailed below except that measurements were performed for individual samples (n = 3).

Experiment 6 (Figure 4 and S5): We tested the single strains and both SynComs, each with 5 replicates, for whether they could use MBOA as sole carbon source for growth. We followed the same procedure as described above in Experiment 1 but used minimal media (described previously (4)) containing MBOA at concentrations of 500 and 2'500 µM. As positive controls for growth, we grew the bacteria in minimal medium supplemented with glucose (500 and 2'500 µM) as sole carbon source. We continuously recorded the OD_{600} of the cultures and stopped the experiment at 68 h. For a measure of bacterial growth, we calculated the area under the growth curve (AUC, x-axis for time and y-axis for OD_{600}) of the OD_{600} readings using the function *auc()* from package MESS (3) in R. We normalized growth in each treatment relative to the control. See analysis scripts for details.

Experiment 7 (Figure 5, S6 and S7): For scale reasons, this experiment was performed in Erlenmeyer flasks and not in 96-well plates as the other experiments. Modifications included that the strains were pre-cultured overnight in 50 ml Erlenmeyer flasks containing 30 ml liquid 50% TSB and then mixed in equal ratios (OD_{600} = 0.6) as nonSC and metSC SynComs. The assembled SynComs were pelleted (5 min at 3'600 rpm), washed twice with 10 mM $MgCl_2$ buffer (Sigma-Aldrich, St. Louis, USA) and diluted in 35 mL of 10 mM $MgCl_2$. Samples of these 'input' SynComs (i.e. SynComs at the start of the experiment when inoculating the Erlenmeyer flasks) were aliquoted in 1.5 ml microcentrifuge tubes (5 replicates) and stored at -80 °C for community analysis. The same concentration of 10 mM $MgCl_2$ was added to the control treatment (NBC). Analogous to the other Experiments, cultures were grown in liquid 50% TSB supplemented with either 500 or 2'500 µM of MBOA or DMSO as a control. Five replicate cultures were set up for each of the 6 sample groups (2 SynCom * 3 treatments). The experiment was stopped at 68 h and the OD_{600} of each culture was recorded using a biophotometer (Eppendorf, Hamburg, Germany). Of each sample group, aliquots were fixed for metabolite analysis (details below). Metabolite measurements (n = 5) were made on five replicate cultures per sample group. We also collected samples for community analysis by 16S rRNA gene amplicon sequencing (see below). For each of the 5 replicates per sample group, we collected two samples of the same culture resulting in n=10 replicates for community analysis. The samples were stored at -80 °C until processing. Finally, we assessed community size for each replicate (n=5) per sample group based on colony forming units (CFU) determined by plating serial dilutions on TSA plates and incubating them at 22 °C.

Metabolite analysis from bacterial cultures

Samples for metabolite analyses were processed and measured as described previously (2). To fix the metabolites in the bacterial cultures, we added 150 µL of the cultures to 350 µL of extraction buffer (100% methanol (MeOH) + 0.14% formic acid (FA)) in non-sterile round bottom 96-well plates (Thermo Fisher Scientific, Waltham, USA). We stored the fixed samples with a final concentration of 70% MeOH and 0.1% FA at -80 °C. To reduce the number of samples, we pooled replicates of the same sample group unless otherwise noted. We diluted the pooled sample by adding 50 to 700 µL MeOH 70% + 0.1% FA and filtered the cultures through regenerated cellulose membrane filters (CHROMAFIL RC, 0,2 µm; Macherey-Nagel, Düren, Germany) by centrifugation (3'220 g for 2 min) to remove bacterial debris. To pellet any residual particles, we centrifuged the extracts at 11'000 g for 10 min at 4 °C. We aliquoted the supernatants in analytical glass vials (Screw Neck Vials, 1 ml; VWR, Dietikon, Switzerland) and stored the samples at -20 °C until analysis.

We profiled the benzoxazinoids and their degradation products in the fixed and filtered extracts of the bacterial cultures using an Acquity I-Class UHPLC system (Waters, Milford, US) coupled to a Xevo G2-XS QTOF mass spectrometer (Waters, Milford, US) equipped with a LockSpray dual electrospray ion source (Waters, Milford, US). Gradient elution was performed on an Acquity BEH C18 column (2.1 x 100 mm i.d., 1.7 mm particle size; Waters, Milford, US) at 98–50% A over 6 min, 50-100% B over 2 min, holding at 100% B for 2 min, re-equilibrating at 98% A for 2 min, where A = water + 0.1% FA and B = acetonitrile + 0.1% FA. The flow rate was 0.4 mL/min. The temperature of the column was maintained at 40 °C, and the injection volume was 1 µL. The QTOF MS was operated in sensitivity mode with a positive polarity. The data were acquired over an m/z range of 50–1'200 with scans of 0.1 s at a collision energy of 6 V (low energy) and a collision energy ramp from 10 to 30 V (high energy). The capillary and cone voltages were set to 2 kV and 20 V, respectively. The source temperature was maintained at 140°C, the desolvation temperature was 400 °C at 1'000 L/hr and the cone gas flow was 100 L/hr. Accurate mass measurements (<2 ppm) were obtained by infusing a solution of leucine encephalin at 200 ng/mL at a flow rate of 10 µL/min through the Lockspray probe (Waters, Milford, US). For each expected benzoxazinoid, standard compounds with four concentrations were run together with the samples (DIMBOA-Glc, DIMBOA, HMBOA, MBOA-Glc, MBOA, BOA, AMPO, APO, AAMPO, HMPMA, each at 10, 50, 200, and 400 ng/mL; HMPAA at 40, 200 ng/mL, 1 and 10 µg/mL). For experiment 3 and 5, no standards were run together with the samples. Here in this work, we only report MBOA, AMPO, AAMPO and HMPAA. The compound peaks in the raw chromatogram data were integrated using MassLynx 4.1 (Waters, Milford, US) and identified based on the reference compounds in the standards. Further, we searched for candidate

compounds increasing in metSC over time using Progenesis QI Software (Waters, Milford, USA) and analyzed it in R statistical software. All source data and R code used for graphing are available from https://github.com/PMI-Basel/Thoenen_et_al_SynCom.

DNA extraction, library prep and sequencing

The DNA was extracted from two technical replicates per culture using the NucleoSpin Soil kit (Macherey-Nagel, Düren, Germany). For DNA extraction, 1 mL of culture was pelleted at 13'000 rpm, then the first buffer was directly added to the pellet. Afterwards the extraction was performed following the manufacturer's instructions. The DNA concentration was quantified with the AccuClear® Ultra High Sensitivity dsDNA Quantitation Kit (Biotium, Fremont, USA). DNA was diluted to 0.2 ng/μL for subsequent amplification. A two-step PCR was performed, where with first PCR the DNA is amplified and in the second PCR the PCR products are tagged with custom barcodes. For the first PCR, the 16S rRNA gene was amplified with the specific primers 515-F (GTGYCAGCMGCCGCGGTAA, (5) and 806*-R (TTAGAWACCCBNGTAGTCC). The 806*-R was shortened by one base to avoid mismatch with the 16S sequence of the Microbacteria strains. The PCR reaction mix was composed of 5 μL DNA template, 0.4 μL of 10 μM 515-F and 806*-R primers each, 8 μL 5Prime HotMasterMix (Quantabio, Beverly, USA), 2 μL 3% BSA and 4.6 μL autoclaved MilliQ water to a final reaction volume of 20 μL. The cycling profile was 94°C for 3 minutes, 25 cycles of 94°C for 45 seconds, 60°C for 60 seconds, 72°C for 90 seconds, and 72°C for ten minutes. All PCR reactions were purified with SPRIselect beads (Beckman Coulter Life Sciences, Indianapolis, USA) following manufacturer's instructions. The reaction products from the first PCR were next amplified for another 10 cycles with uniquely barcoded primer pairs for each sample in a second PCR. The barcoded PCR products were purified with SPRIselect beads, DNA was quantified with the Qubit™ dsDNA BR kit (Invitrogen, Thermo Fisher Scientific, Waltham, MA, USA), and the samples were pooled equimolar using a Myra Liquid Handler (Bio Molecular Systems, Upper Coomera, Australia). The pooled library was bead purified and sequenced by MiSeq v2 500 cycle nano sequencing kit at the Next Generation Sequencing Platform (University of Bern) using the 2x 250 bp pair-end sequencing protocol (Illumina Inc., San Diego, USA).

Community analysis details

The raw sequencing data was processed in R using the DADA2 pipeline version 1.20 (6). The analysis steps were wrapped in a Snakemake pipeline (7). In brief, raw reads were filtered and trimmed with the function *filterAndTrim()*. This step trims the 19 bp long primer sequences (trimLeft and trimRight = 19) and discards low-quality reads (truncQ = 2), reads matching PhiX and reads containing Ns. The reads were then subjected to the error learning step using *learnErrors()*, dereplicated using *derepFastq()* and denoised using dada (derepFs, err=errF, multithread=TRUE) following the DADA2 pipeline with default options. Next, the read pairs were

merged using *mergePairs()* (minimal overlap of 20 bp) and chimera were removed using *removeBimeraDenovo()*. Finally, taxonomic assignment of the high-quality sequences was done against the SILVA database (v128; (8) using *assignTaxonomy()* and *addSpecies()* functions. A phyloseq object was exported for downstream analysis. **Dataset S2** documents all the dada2 pipeline. The source code for the analysis of the raw sequencing data is available from https://github.com/makrez/Analysis_documentation_Synthetic_communities_mrb.

For analysis of the community data, we first mapped the 16S rRNA gene sequences of the SynCom members (all MRB strains have Sanger sequences available; (1) to the amplicon sequences using usearch (9) with an identity of 0.97. ASVs mapping to the SynCom members were used for calculation, other low abundant and non-mapping ASVs were discarded. The counts of the community data were normalized by rarefaction using phyloseq (10) and the abundances of the strains were further adjusted based on their estimated copy numbers of the 16S rRNA genes (**Dataset S1**). Compositional differences between the SynComs and by the treatments were tested with Permutational Analysis of Variance (PERMANOVA, 99999 permutations; model: ~ SynCom * Treatment) on Bray Curtis distances using the R package vegan (11). The effects on community composition using the same model were visualized with a Canonical Analysis of Principal coordinates (CAP) using the R package phyloseq (10). All source data and code used for statistical analysis and graphing are available from https://github.com/PMI-Basel/Thoenen_et_al_SynCom.

Supplementary Results

Enterobacter LMX9231

Enterobacter LMX9231, one of the six core bacteria of both SynComs, needs more detailed explanations. On solid media, LMX9231 had been identified earlier to color MBOA-containing agar red during 10 days of growth, indicating AMPO formation (**Fig. S2A**, (2). In liquid culture however, LMX9231 is not an efficient degrader of MBOA. Compared to *Microbacterium* LMB2, which fully degraded MBOA, LMX9231 only partially degraded MBOA in 68 h of cultivation both in 96-well plates (**Fig. S2B**) or in shake flasks (**Fig. S2C**). It is important to note that LMX9231 can form AMPO, but it does not fully degrade MBOA to AMPO as LMB2 in the tested conditions. Throughout the study, LMX9231‘s weak ability to degrade MBOA did not compromise the non-metabolizing SynCom characteristic, as the nonSC never markedly degraded MBOA (see **Figs. 1B, S4A** and **S6AB**).

It appears plausible that *Enterobacter* LMX9231 may possess an alternative and less efficient pathway for AMPO formation than LMB2. When we found BxdA, we noticed that the best homologies in Enterobacteriaceae showed less than 30% similarity to BxdA on amino acid level compared to BxdA of efficient AMPO formers belonging to the Microbacteriaceae and Sphingomonadaceae (**Fig. S2D**). This low level of protein similarity of Enterobacteriaceae was consistent with all other tested strains that did not show strong AMPO-formation in liquid culture. We therefore think, that Enterobacteriaceae may possess another, BxdA-independent mechanism responsible for the AMPO-formation and this pathway seems to operate slower (10d of growth) on Agar-plates and less efficient in liquid cultures.

Supplementary Figures

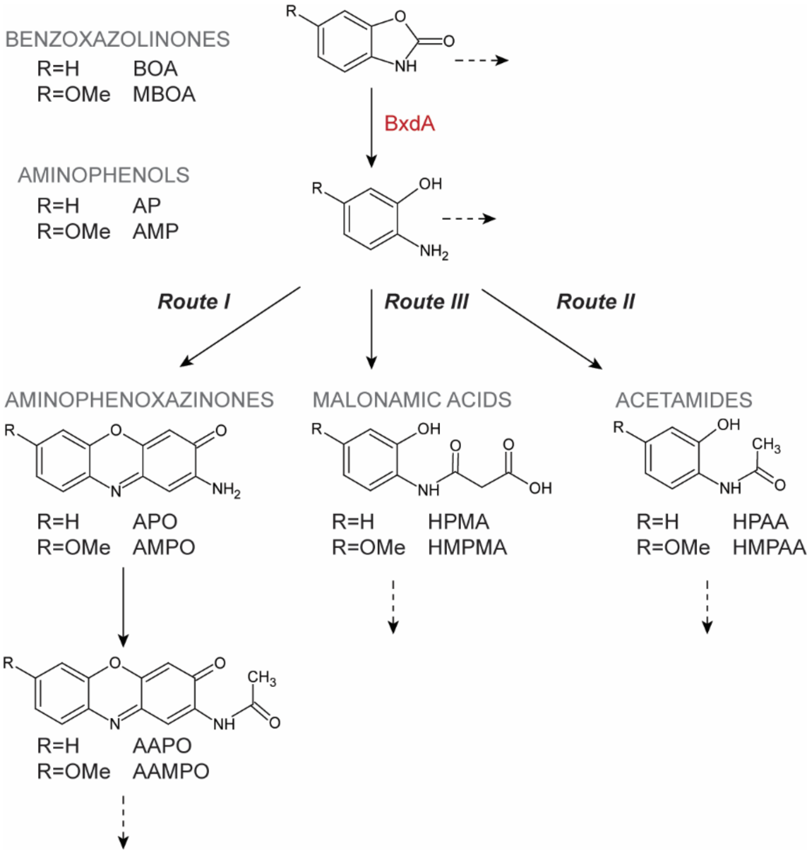

Abbreviation	Full name	Class	Mass [g/mol]	Formula
DIMBOA-Glc	2-O-β-D-glucopyranosyl-2,4-dihydroxy-7-methoxy-(2H)-1,4-benzoxazin-3(4H)-one	Benzoxazinone glucoside	373.31	C15H19NO10
DIMBOA	2,4-dihydroxy-7-methoxy-1,4-benzoxazin-3-one	Benzoxazinone aglucone	211.17	C9H9NO5
MBOA	6-methoxybenzoxazolin-2(3H)-one	Benzoxazolinone	165.15	C8H7NO3
BOA	Benzoxazolin-2-(3H)-one	Benzoxazolinone	135.10	C7H5NO2
AMP	2-amino-5-methoxyphenol	Aminophenol	139.15	C7H9NO2
AP	2-aminophenol	Aminophenol	109.13	C6H7NO
HPAA	2-acetamidophenol	Acetamide	151.16	C8H9NO2
HMPAA	N-(2-hydroxy-4-methoxyphenyl)acetamide	Acetamide	181.19	C9H11NO3
HPAA	N-(2-hydroxyphenyl)acetamide	Acetamide	151.16	C8H9NO2
HMPMA	N-(2-hydroxy-4-methoxyphenyl)malonamic acid	Malonamic acid	225.2	C10H11NO5
HPMA	N-(2-hydroxyphenyl)malonamic acid	Malonamic acid	195.17	C9H9NO4
AMPO	2-amino-7-methoxy-phenoxazin-3-one	Aminophenoxazinones	242.23	C13H10N2O3
APO	2-amino-(3H)-phenoxazin-3-one	Aminophenoxazinones	212.20	C12H8N2O2
AAMPO	2-acetylamino-7-methoxy-phenoxazin-3-one	Aminophenoxazinones	284.27	C15H12N2O4
AAPO	2-acetylamino-(3H)-phenoxazin-3-one	Aminophenoxazinones	254.24	C14H10N2O3

Supplementary Figure S1: Benzoxazinoid and benzoxazolinone metabolites of this study. Benzoxazinoid metabolites are produced by plants and the benzoxazolinone degradation pathway by microbes takes place in soil. The table lists the full chemical name, the compound class, the molar mass and the chemical formula. Dashed arrows refer to possible further metabolisation routes.

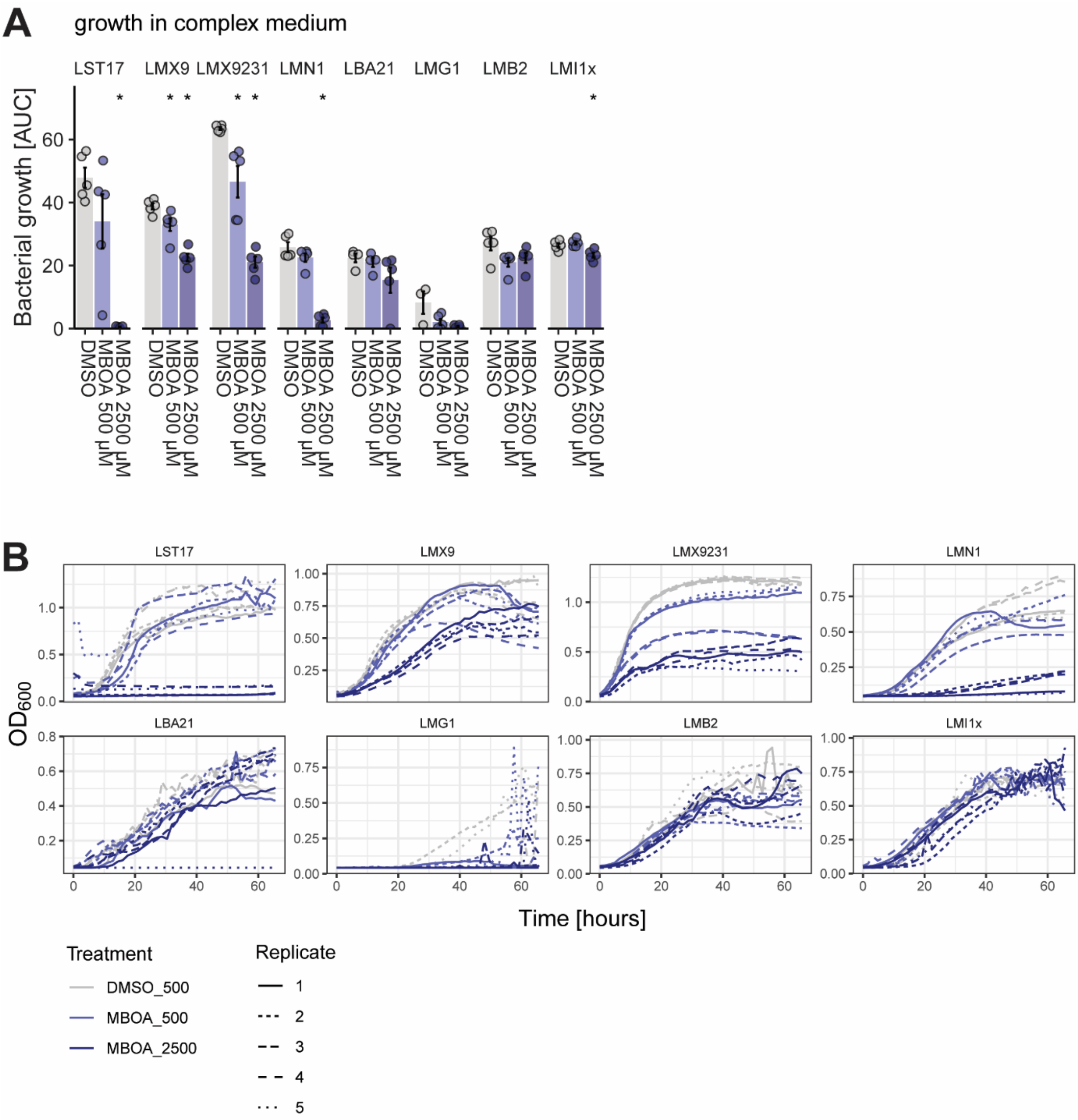

Supplementary Figure S2: Growth of individual SynCom members in presence of MBOA. Bacterial growth was assessed in 50% TSB supplemented with MBOA (500 and 2'500 µM) or DMSO only (negative control). Five replicates were grown for each strain (n = 5). **A)** Means ± standard errors are reported and asterisks indicate significant differences between treatments and DMSO controls (pairwise t-test, Bonferroni-adjusted $P < 0.05$). **B)** Absorbance over time (OD600).

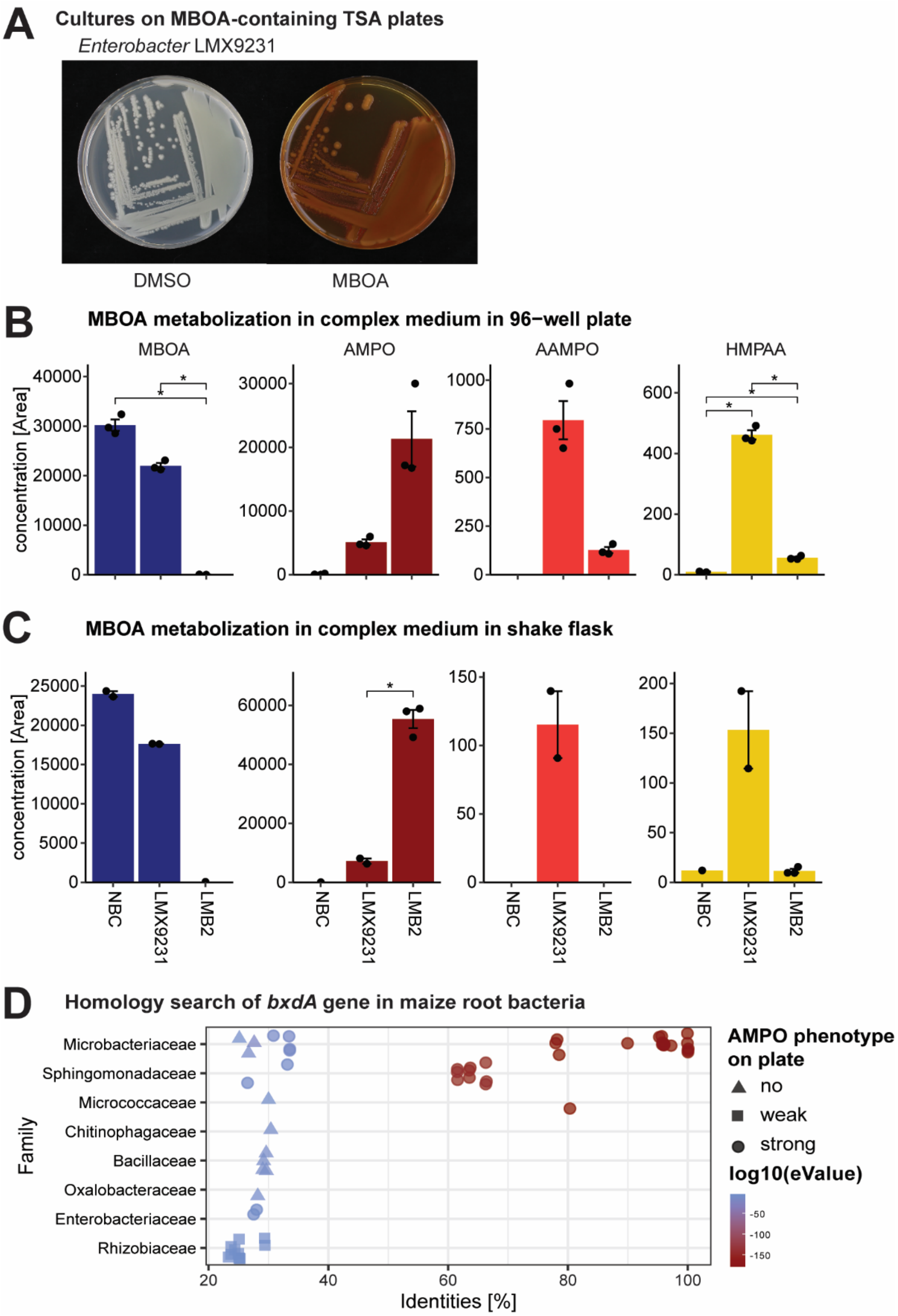

Supplementary Figure S3: MBOA metabolization phenotype of *Enterobacter* LMX9231: A) Pictures of *Enterobacter* LMX9231 grown on 100% TSA plates containing DMSO (2 mL/L) or MBOA (200 mg/L; ~1'200 µM) for 10 days suggest AMPO formation. **B, C)** Metabolization of MBOA and benzoxazinoid metabolites formed by *Enterobacter* LMX9231 and *Microbacterium* LMB2 (positive control) in 50% TSB supplemented with 500 µM MBOA **B)** in 96-well-plates and **C)** shake flasks. **D)** Homology searches with the protein BxdA from *Microbacterium* LMB2 across all genome sequenced strains of the MRB collection. The panel **D** of this figure was shown in a previous publication (2).

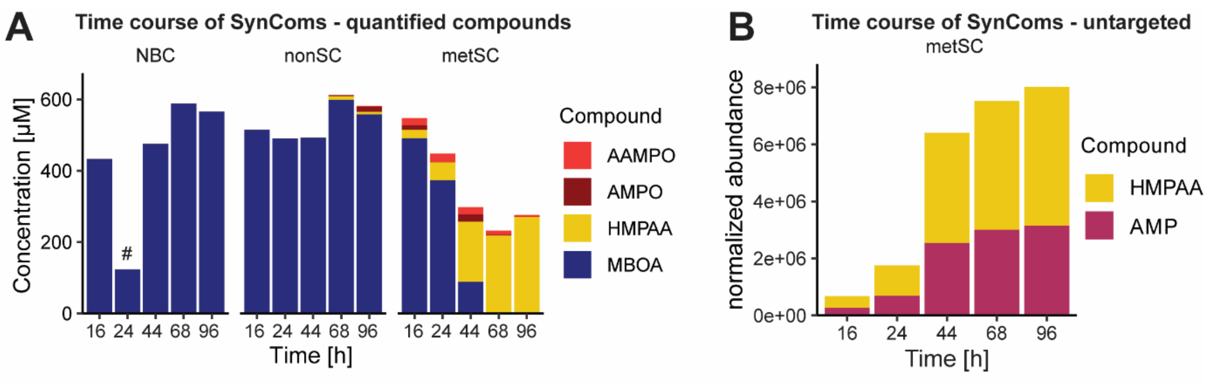

Supplementary Figure S4: Metabolization of MBOA by SynComs. The nonSC and metSC SynComs were grown in 96-well plates containing 50% TSB supplemented with 500 µM MBOA and samples were collected at the indicated time points up to 96 h. The 68 h time point is shown in Fig. 1B. **A)** The concentrations of MBOA, AMPO, AAMPO and HMPAA metabolites were quantified using analytical standards while in **B)** the intermediate AMP (see Fig. S1) is reported based on the normalized peak area (peak corresponding to the mass of AMP). HMPAA is reported again but on its normalized peak area for reference. Metabolite measurements (n = 1) were made on pools of three independently grown cultures (#: sample with failed pooling). Part of panel **A** of this figure (NBC) was shown in a previous publication (2).

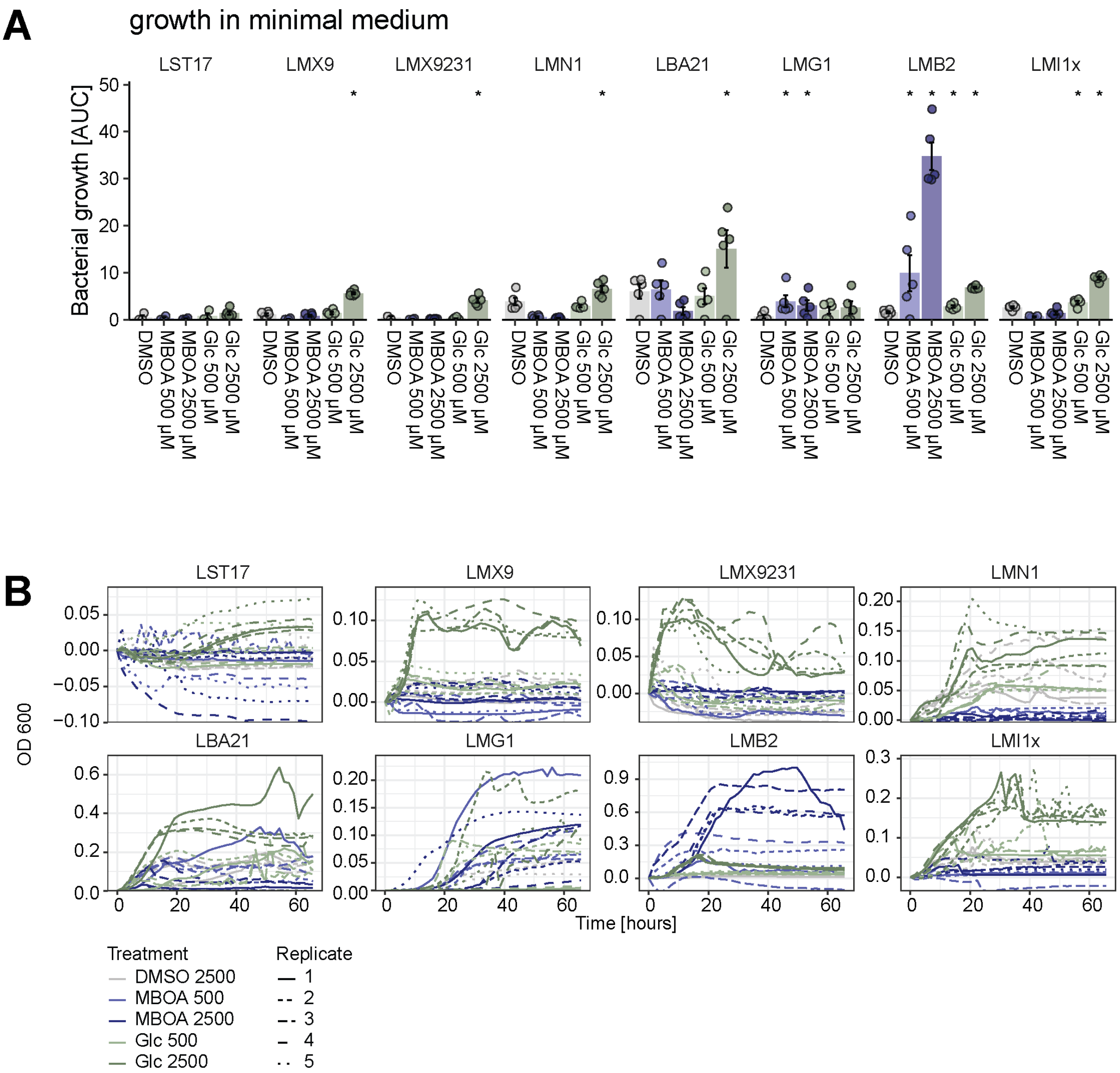

Supplementary Figure S5: Growth of individual SynCom members in minimal media. Bacterial growth in the minimal medium with DMSO (negative control), MBOA (500 and 2’500 µM) or glucose (positive control; same concentrations) as sole carbon sources. Five replicates were grown for each strain (n=5). **A)** Means ± standard errors are reported and asterisks indicate significant differences against their DMSO control (pairwise t-test, Bonferroni-adjusted P < 0.05). **B)** Absorbance over time. Part of the data of this figure was shown in a previous publication (2).

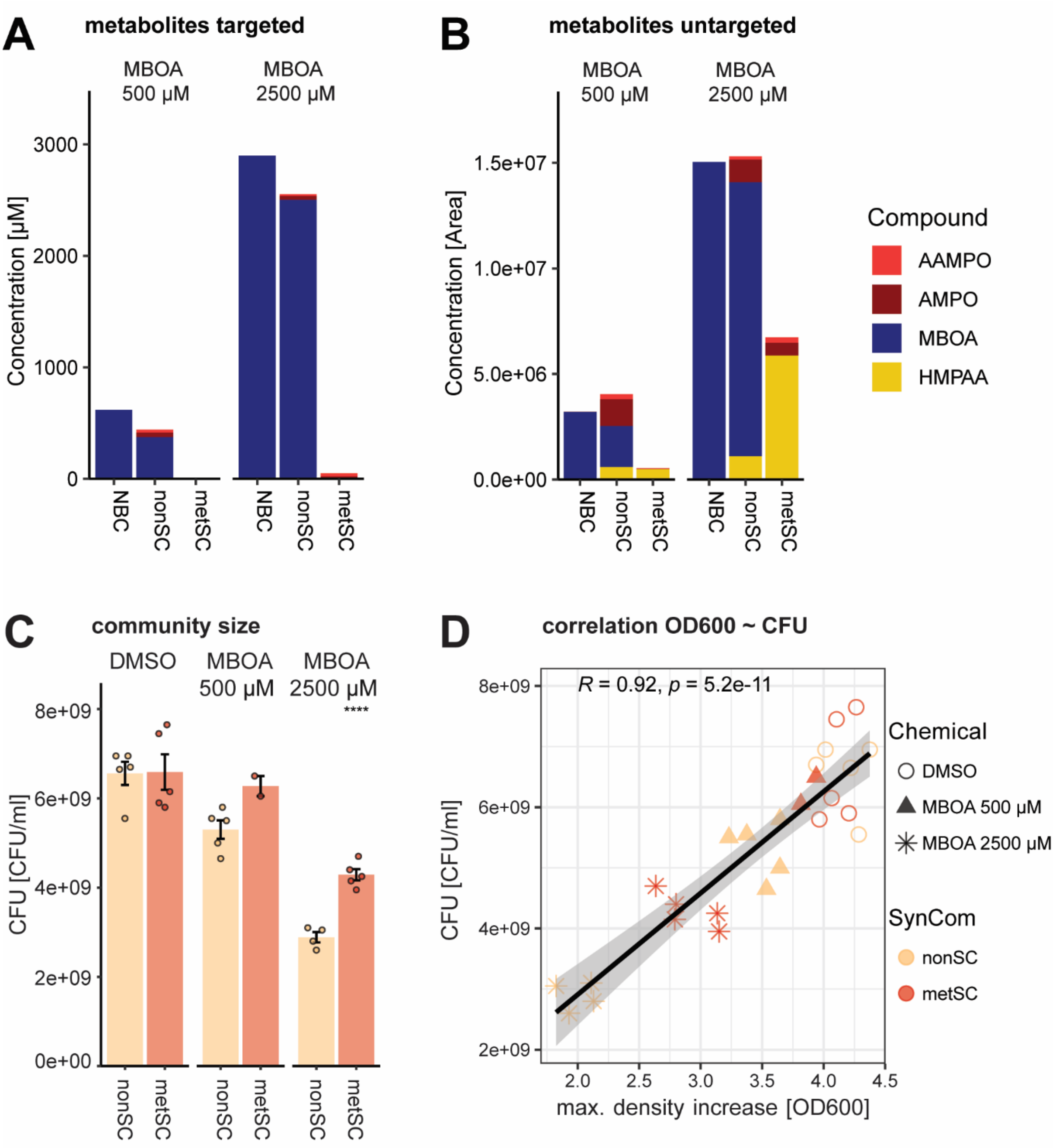

Supplementary Figure S6: Metabolites and growth of the SynComs in presence and absence of MBOA. SynComs were grown in shake flasks containing 20 mL 50% TSB supplemented with DMSO or 500 µM or 2’500 µM MBOA until harvest at 68 h. Five replicate cultures were set up for each SynCom and treatment. **A)** Measurements of metabolites after exposure of both SynComs and their no bacteria control (NBC, media with MBOA but without bacteria) to MBOA. Shown are stacked barplots of means of the different metabolites (n=5). Note that HMPAA is not shown in A because of absence of a standard. **B)** Untargeted measurements of metabolites, relative concentrations were calculated from the area of the integrated peaks. **C)** Community size was determined by counting the colony forming units (CFU/mL) at the end of the experiment (n=5). Asterisks indicate significant differences between the SynComs (pairwise t-test, Bonferroni-adjusted $P < 0.05$). **D)** Correlation of the two measurements for bacterial growth, OD_{600} compared to CFU counts (shown in Fig. 4A).

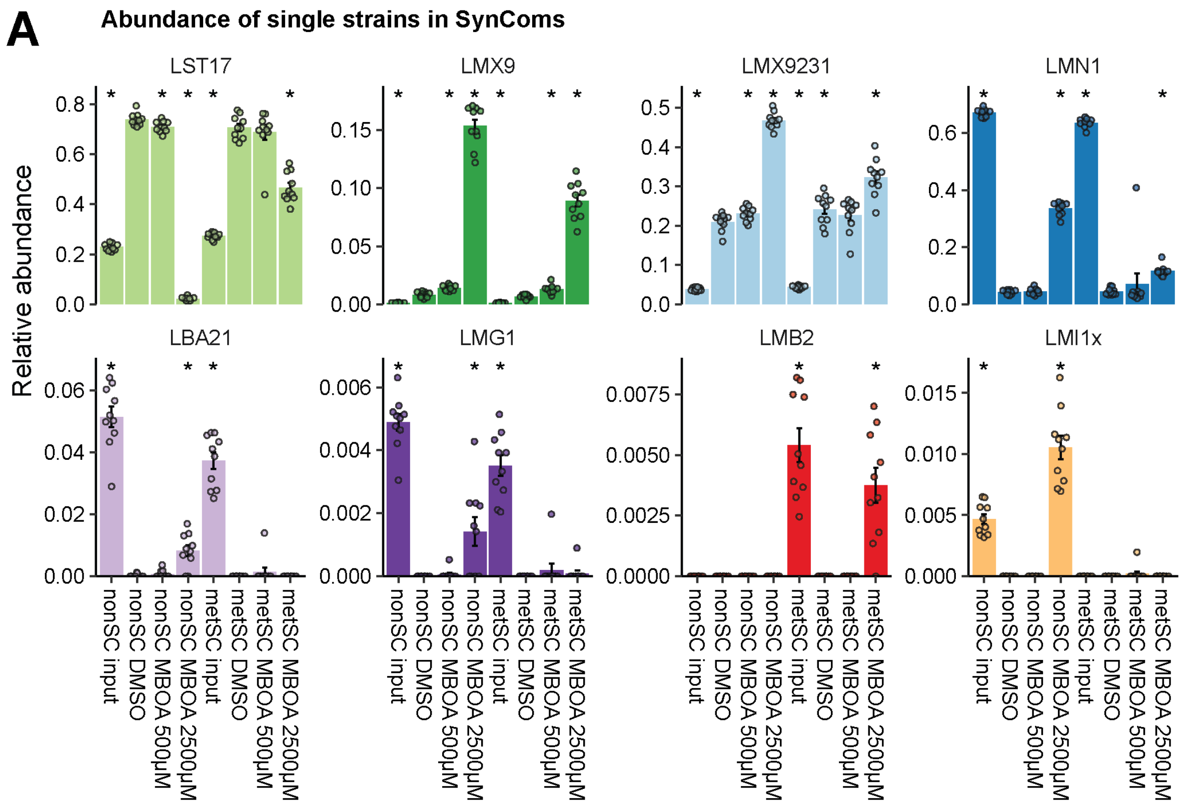

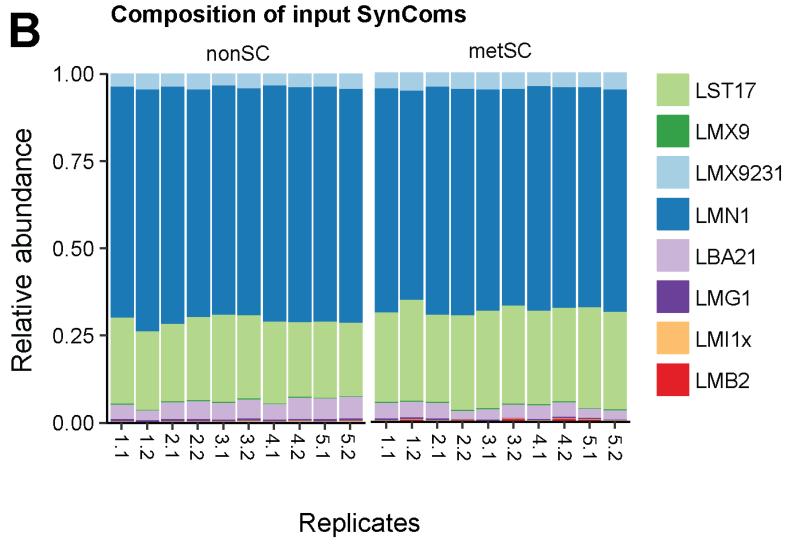

Supplementary Figure S7: Abundance of single strains in the SynComs in presence and absence of MBOA. SynComs were grown in shake flasks containing 20 mL 50% TSB supplemented with DMSO or 500 µM or 2500 µM MBOA until harvest at 68 h. Five replicate cultures were set up for each SynCom and treatment and two parallel samples of the same culture were collected for community analysis by 16S rRNA gene amplicon sequencing. **A)** Relative abundances of each single strain in the SynCom at the beginning of the experiment (input) and treatment combinations during the experiment. Asterisks indicate significant differences between the treatments (pairwise t-test, Bonferroni-adjusted $P < 0.05$). **B)** Community analysis was also conducted with the 'input' SynComs, i.e. to determine the strain abundances at the start of the experiment.

Supplementary Tables

Table S1: PERMANOVA of SynCom composition

Factorial effects on community compositions were assessed using PERMANOVA based on Bray Curtis distances and the model ~SynCom * Treatment (n=10).

	Df	SumOfSqs	R^2	F	Pr(>F)
**Treatment**	2	0.7837848	0.2598361	10.467239	**0.001**
**SynCom**	1	0.0504273	0.0167174	1.346886	0.26
**Treatment:SynCom**	2	0.1604922	0.0532055	2.14333	0.063
**Residual**	54	2.0217547	0.6702411	NA	NA
**Total**	59	3.0164589	1	NA	NA

Supplementary Datasets

Dataset S1: Table contains information on all bacterial strains used in the SynCom, taxonomy information, 16S sanger sequence and the number of 16S copies.

Dataset S2: Documentation of dada2 pipeline script used. This file is a html markdown of the R script containing the code to process the raw sequencing reads from the community profiling (Experiment 5) to the phyloseq object which was then further analyzed in R.

References

1. Thoenen L, Giroud C, Kreuzer M, Waelchli J, Gfeller V, Deslandes-Hérold G, et al. Bacterial tolerance to host-exuded specialized metabolites structures the maize root microbiome. Proc Natl Acad Sci. 2023 Oct 31;120(44):e2310134120.

2. Thoenen L, Kreuzer M, Pestalozzi C, Florean M, Mateo P, Züst T, et al. The lactonase BxdA mediates metabolic specialisation of maize root bacteria to benzoxazinoids. Nat Commun. 2024 Aug 2;15(1):6535.

3. Ekstrøm C. MESS: Miscellaneous Esoteric Statistical Scripts. 2016.

4. Peyraud R, Kiefer P, Christen P, Massou S, Portais JC, Vorholt JA. Demonstration of the ethylmalonyl-CoA pathway by using 13C metabolomics. Proc Natl Acad Sci U S A. 2009 Mar 24;106(12):4846–51.

5. Ul-Hasan S, Bowers RM, Figueroa-Montiel A, Licea A, Licea-Navarro AF, Beman JM, et al. Community ecology across bacteria, archaea and microbial eukaryotes in the sediment and seawater of coastal Puerto Nuevo, Baja California. PLOS ONE. 2019 Feb 14;14(2).

6. Callahan BJ, McMurdie PJ, Rosen MJ, Han AW, Johnson AJA, Holmes SP. DADA2: High-resolution sample inference from Illumina amplicon data. Nat Methods. 2016 Jul;13(7):581–3.

7. Mölder F, Jablonski KP, Letcher B, Hall MB, Tomkins-Tinch CH, Sochat V, et al. Sustainable data analysis with Snakemake. F1000Research. 2021;10:33.

8. Callahan B. Silva Taxonomic Training Data Formatted For Dada2 (Silva Version 128). 2017 Jan 1;

9. Edgar RC. Search and clustering orders of magnitude faster than BLAST. Bioinformatics. 2010 Oct 1;26(19):2460–1.

10. McMurdie PJ, Holmes S. phyloseq: an R package for reproducible interactive analysis and graphics of microbiome census data. PLOS ONE. 2013 Apr 22;8(4).

11. Oksanen J, Blanchet F, Friendly M, Kindt R, Legendre P, McGlinn D, et al. vegan: Community Ecology Package. 2019.
